# Role of Key Salt Bridges in Thermostability of *G. thermodenitrificans* EstGtA2: Distinctive Patterns within the New Bacterial Lipolytic Enzyme Family XV

**DOI:** 10.1371/journal.pone.0076675

**Published:** 2013-10-08

**Authors:** David M. Charbonneau, Marc Beauregard

**Affiliations:** 1 Département de chimie-physique, Centre de recherche sur les matériaux lignocellulosiques, Université du Québec à Trois-Rivières, Trois-Rivières, Québec, Canada; 2 PROTEO (Quebec network for research on protein structure, function and engineering), Université Laval, Québec, Québec, Canada; University of Florida, United States of America

## Abstract

Bacterial lipolytic enzymes were originally classified into eight different families defined by Arpigny and Jaeger (families I-VIII). Recently, the discovery of new lipolytic enzymes allowed for extending the original classification to fourteen families (I-XIV). We previously reported that *G. thermodenitrificans* EstGtA2 (access no. AEN92268) belonged to a novel group of bacterial lipolytic enzymes. Here we propose a 15^th^ family (family XV) and suggest criteria for the assignation of protein sequences to the N’ subfamily. Five selected salt bridges, hallmarks of the N’ subfamily (E3/R54, E12/R37, E66/R140, D124/K178 and D205/R220) were disrupted in EstGtA2 using a combinatorial alanine-scanning approach. A set of 14 (R/K→A) mutants was produced, including five single, three double, three triple and three quadruple mutants. Despite a high tolerance to non-conservative mutations for folding, all the alanine substitutions were destabilizing (decreasing *T*_*m*_ by 5 to 14°C). A particular combination of four substitutions exceeded this tolerance and prevents the correct folding of EstGtA2, leading to enzyme inactivation. Although other mutants remain active at low temperatures, the accumulation of more than two mutations had a dramatic impact on EstGtA2 activity at high temperatures suggesting an important role of these conserved salt bridge-forming residues in thermostability of lipolytic enzymes from the N’ subfamily. We also identified a particular interloop salt bridge in EstGtA2 (D194/H222), located at position *i* -2 and *i* -4 residues from the catalytic Asp and His respectively which is conserved in other related bacterial lipolytic enzymes (families IV and XIII) with high tolerance to mutations and charge reversal. We investigated the role of residue identity at position 222 in controlling stability-pH dependence in EstGtA2. The introduction of a His to Arg mutation led to increase thermostability under alkaline pH. Our results suggest primary targets for optimization of EstGtA2 for specific biotechnological purposes.

## Introduction

The understanding of sequence-encoded information has never been as important as in this post-genomic era where protein purification and characterization is dramatically outpaced by the volume of DNA sequences deposited in various databanks. Our inability to predict protein behaviour using sequence information drastically slows the pace of discovery. By improving our understanding of sequence features that control enzymatic properties or permit enzyme assignment to a characterised group of related enzymes, we will achieve more accurate extraction of protein character information from genomic data.

Carboxylesterases (EC 3.1.1.1) and lipases (EC 3.1.1.3) are lipolytic enzymes belonging to the class of hydrolases which catalyze the hydrolysis or synthesis of ester bonds in lipids. They adopt the α/β hydrolase fold and bear a catalytic triad formed by Ser-Asp/Glu-His, but differ in term of substrate specificity [1,2]. Carboxylesterases catalyze the hydrolysis of ester bonds in short-chain and partially water-soluble substrates while lipases prefer long-chain triglycerides. They are widely distributed in nature from prokaryotes to mammals and involved in many physiological roles including carbon utilization, detoxification and processing of neurotransmitters to name but a few examples. Prokaryotic lipolytic enzymes have been assigned to eight different families (I-VIII) based on sequence identity and biochemical properties by Arpigny and Jaeger [3]. Triacylglycerol lipases are grouped in family I, while carboxylesterases are grouped in families II-VIII. Recently, the discovery of new bacterial lipolytic enzymes led to the establishment of new families that diverge from the original 8-family classification (families IX-XIV) [4]. The fact that Est30 (CE_GK_) from *Geobacillus kaustophilus* (99% identity with Est30 from *G. stearothermophilus*, PDB code. 1TQH) did not fit in the 8-family classification was reported [5,6]. It was argued that CE_GK_ was different from families IV and VI on the basis of not only the low sequence identity, but also on the lid/cap structures and the number of strands in the core β-sheet. The Est30-like enzymes form the family XIII. We previously suggested that the novel *Geobacillus thermodenitrificans* EstGtA2 diverged from the classification proposed by Arpigny and Jaeger, and was instead a representative of a new family of thermostable lipolytic enzymes named N’ [7]. Despite important similarities between N’ and Est30-like enzymes (family XIII), the N’-related enzymes form a clearly distinct group including monoacylglycerol lipases. A similar conclusion was reached for LipS (from *Symbiobacterium thermophilum*), [8]. Interestingly, an earlier, inclusive classification by Fischer allows assigning Est30-like enzymes (family XIII) to family abH11.01 (Fischer et al 2003). Similarly, the N’ group can be assigned to a cluster included in family abH11.03. The recently proposed LipS group would also belong to abH11.03 family as proposed by Fischer et al [9]. Here we will revisit the various classifications or family names suggested for these lipolytic enzymes and attempt to reconcile them by proposing their assignment to the new 15^th^ family (family XV), named chronologically based on the extension of the Arpigny and Jaeger’s classification.

Thermal stability of lipolytic enzymes has become a practical concern as a result of their applications in different biotechnological processes [10]. Due to their high thermal stability, carboxylesterases and lipases from thermophiles have been and are still intensively studied. Considerable efforts have been made to understand mechanisms involved in determining protein thermostability. Electrostatic interactions between opposite charge residues have gained attention as important determinants of thermostability in thermophilic proteins, which often exhibit more salt bridges or charged networks than their mesophilic counterparts [11-13]. Multiple salt bridges are accepted as one of the most important features that contribute to enzyme thermostability. A statistical analysis of 18 non-redundant families of thermophilic and mesophilic proteins revealed similar hydrophobicities, compactness, polar and non-polar contributions to surface areas, main-chain and side-chain hydrogen bonds. At variance with such conservation of features, the number of salt bridges and side-chain-side-chain hydrogen bonds increased in the majority of thermophilic proteins. In addition, comparison of thermophilic and mesophilic homologous proteins indicated that arginine and tyrosine are significantly more frequent in thermophilic versions [14].

Although many studies have suggested a potential impact of surface electrostatic interactions on protein thermostability, the precise contribution of surface salt bridges to protein stability has long been disputed and is still debated [15-18]. Since formation of a salt bridge depends on the pH of the environment and on the p*K*_a_ of the charged groups involved in the ion pair (-NH_3_^+^ ∙ ∙ · ^-^ OOC-), the contribution of a particular surface salt bridge to thermal stability may vary according to pH conditions. Therefore, depending of the identity of the charged residues involved, the contribution for thermal stability becomes pH-dependent. Other factors are also at play in determining the extent of stability afforded by a bridge [18-20].

Historically, stability determinants have been largely inferred from comparisons of homologous proteins from distant mesophilic vs. thermophilic organisms. Unfortunately, most attempts to use such putative stability determinants in a different context have yielded unpredictable results [21,22]. The overall impact of the introduction of new salt bridges on enzyme thermotolerance can also be negative, depending on the contribution of short- and long-range interactions and other factors [23-26]. Parameters such as level of exposure to solvent, relative orientation of the side-chains, short- and long-range interactions play a determinant role, and may vary widely when comparing distant proteins.

The ability to predict the impact of new salt bridges as stabilization devices in mutant proteins using canonical principles derived from distant proteins has remained elusive. But comparison of closely related proteins with differences in thermostability or other properties should reveal structure-properties relationships of direct relevance for the proteins chosen [27]. In this respect, the high level of identity between enzymes included in the N’ cluster (85-100% identity) makes them ideal subjects for the investigation of conserved structural determinants for enzyme properties, including thermostability.

Here we identified eight salt bridges in EstGtA2 that are highly conserved in the N’ cluster. Of these, five salt bridges involving Arg or Lys residues are strictly conserved and might be useful in as potential sequence indicator for the assignation of newly discovered enzymes to the N’ subfamily. Our results show that these five selected basic residues are involved in controlling thermostabilty in EstGtA2. We also explored the dependence of stability on pH by mutating a particular interloop salt bridge found in proximity to the catalytic site. Our work on EstGtA2 opens vista on structural determinants for thermostability of lipolytic enzymes from the N’ subfamily.

## Materials and Methods

### Strains and plasmids

The EstGtA2 ORF (GenBank accession no. JN031579) was amplified by PCR from *G. thermodenitrificans* strain CMB-A2 (GenBank accession no. GQ293454) [7], and cloned into BL21-Gold(DE3)pLysS strain: *E. coli* B F ^-^ *omp*T *hsd*S(r_B_^–^ m_B_^–^)*dcm* ^+^ Tet ^r^ *gal* λ(DE3) *end*A Hte [pLysS Camr] (Stratagen) using the pET28(a+) expression vector (Novagen).

### Site-directed mutagenesis

Mutations were introduced in EstGtA2 using the QuickChangeII site-directed mutagenesis kit (Stratagen). PCR were carried out with the *pfu* ultra HF DNA polymerase (Stratagen). The mutated PCR products were treated with *Dpn*I and cloned into *E. coli* BL21 using heat shock transformation. Transformed cells were selected on LB-agar containing 50 µg/mL kanamycin. Selected clones were picked and grown in 5 mL LB 50 µg/mL kanamycin overnight at 37°C with agitation (200 rpm). The plasmids were extracted and purified from 3 mL cultures using the Miniprep kit (Qiagen) and 1 ml of each culture was stocked in 20% glycerol and stored at -80°C. Sequencing was carried out using the T7 terminator primer at the Biomolecular platform, Université Laval (Québec). Specified mutated plasmids were used as template at each step for combination of mutations, and additional site-directed mutagenesis. The primers used for cloning and directed mutagenesis are listed in Table S1 in File S1.

### Protein expression and purification

Transformed cells with genes coding for wild type and mutant proteins were grown overnight in 5 mL LB containing 50 µg/mL kanamycin for 16h at 37°C with agitation at 175 rpm. Volumes of 125 mL LB (50 µg/mL kanamycin) were inoculated with 0.125 mL of the respective pre-cultures and were grown at 37 °C until OD_600_^≈^ 0.6 and then induced with 0.5 mM IPTG (final concentration) for 16 h at 20°C with agitation (150 rpm). Cells were harvested by centrifugation and stored at -80°C. Cells were then thawed and ressuspended in 15 mL of lysis buffer (50 mM sodium phosphate, 10 mM imidazole, 150 mM NaCl, pH 7.5 and 1 mg/mL lysozyme was added) and lysed by sonication for 30 sec at 50 pulse s/sec. The lysate was clarified by centrifugation for 30 min at 15 krpm and the expressed His_6_-tagged recombinant proteins were purified from the soluble fraction under native conditions using IMAC (Ni-NTA beads, Qiagen). The nickel resin was washed with 30 mL of buffer. Bound His_6_-tagged proteins were eluted from the nickel resin with 5 mL of elution buffer (20 mM sodium phosphate, 300 mM NaCl, 250 mM imidazole, pH 7.5). Proteins were dialyzed 48h hours at 4°C against 2L of sodium phosphate buﬀer (20 mM), pH 8 using 3,500 kDa MWCO dialysis membranes. The protein concentration was determined by the bicinchoninic acid (BCA) method. The purity of each preparation was verified by SDS-PAGE.

### Enzyme activity measurements

Enzyme activity was assessed in a final volume of 0.2 mL in 96-wells microplates using a Synergy Mx, Biotech microplate reader. Each reaction contained a final concentration of 10 µg/mL (1 µg) of purified protein, with 100 µM (20 nmole) *p*-nitrophenyl octanoate in 20 mM buffer. The buffers used were sodium citrate/citric acid (pH 3-5), sodium phosphate (pH 6-8), Tris-HCl pH9 and CAPS/NaOH (pH 10-12). Specific activity was determined by measuring the amount of paranitrophenol (*p*NP) released after hydrolysis of *p*NP-octanoate at temperatures ranging from 25 to 65°C. The amount of *p*NP produced was recorded by monitoring the absorbance at 405 nm (pH ≥ 7). The absorbance at 340 nm was also monitored under acidic conditions. Standard curves for *p*-nitrophenol absorbance (at each pH and temperature) as function of concentration allowed determining the initial rate of hydrolysis and enzyme specific activity at each pH and temperature. One unit of enzyme activity was defined as the amount of *p*NP (µmole) released by minute per milligram of pure protein. Where specified (H222R and wild type) enzymes were incubated at various temperatures for 10 minutes, then cooled down to 25°C for measurements.

### CD spectroscopy

Far-UV (178-250 nm) and near-UV (250-320 nm) CD spectra were recorded in a Jasco J-720 spectropolarimeter under nitrogen atmosphere with protein concentration of 0.5 mg/mL in 20 mM sodium phosphate pH 8 or CAPS pH 10 using a 0.01 and 1 cm quartz cell respectively. The data were converted into molar ellipticity taking into account the path length and the molar concentration of residues (0.0045 M). For calculation of secondary structures, the far-UV CD data were converted into Δε. The protein secondary structures were calculated using CDSSTR (spectra set no. 3 from 190-260 nm) [28,29]. Thermal unfolding/refolding transitions were measured with a 0.1 cm path length jacketed quartz cell connected to a Neslab RTE-111 circulating water bath containing 50% ethylene glycol. The transitions were monitored by the variation of CD signal at 222 nm between 25 to 90°C upon heating and cooling at a rate of 0.8 °C/min. Transitions were evaluated using a nonlinear least squares fit assuming a two-state model with sloping pre- and post-transitional base lines. The standard errors for *T*_*m*_ calculated from the analysis of the individual melting profiles were smaller than 0.1 °C. Extrapolation and least-square analysis from the pre- and post-transition regions allowed determination of the equations for *y*_*F*_ and*y*_*U*_. Thermal unfolding curves were fitted on a two-state model (F ↔ U). The equilibrium constant was determined at each temperature and the thermodynamic parameters were determined according to the following equations, [30]:

(1) $$f_{U}=(y_{F}-y)/(y_{F}-y_{U})$$

(2) $$K=f_{U}/(1-f_{U})=f_{U}/f_{F}=(y_{F}-y)/(y-y_{U})$$

The free energy change associated with thermal unfolding was calculated at each temperature by the following equation:

(3) $$\Delta G=-RT\ln K=-RT\ln[(y_{F}-y)/(y-y_{U})]$$

The apparent *T*_*m*_ values were then determined by plotting Δ*G* as function of temperature where *T*_*m*_ = *T* at Δ*G* = 0, the midpoint of thermal unfolding curves, where Δ*G*(*T*_*m*_) = 0 = Δ*H*_*m*_ -*T*_*m*_Δ*S*_*m*_. This relation gives parameters to calculate the free energy change at any temperature Δ*G*(*T*), the equation can be given by:

(4) $$\Delta G(T)=\Delta H_{m}(1-T/T_{m})-\Delta C_{P}\left[\left(T_{m}-T)+T\ln(T/T_{m}\right)\right]$$

### Fluorescence spectroscopy

Protein intrinsic fluorescence was measured using a Carry Eclipse spectrofluorimeter with a protein concentration of 0.05 mg/mL in 20 mM phosphate buffer pH 8. For chemical denaturation experiments, 1 mL GuHCl (from 1 to 6 M) was mixed with 1 mL of protein (0.1 mg/mL). Fluorescence was recorded at room temperature with constant protein concentration of 0.05 mg/mL and final GuHCl concentration of 0-3 M in 20 mM sodium phosphate pH 8 using a 1 cm quartz cell.

### In silico analyses

DNA and translated amino acid sequences were edited with Clone Manager Professional Suite version **7.03**. Similarity searches were performed with BLAST 2.0 program [31] and sequences for comparative studies were retrieved from the GenBank database [32] via NCBI Entrez at http://www.ncbi.nlm.nih.gov/Entrez/. Multiple sequence alignments were performed with ClustalW2 [33]. The alignment postscripts were generated using ESPript 2.2 [34]. Phylogenetic analyses were carried out with MEGA 5.1 [35] using the neighbour-joining method [36]. The EstGtA2 3D structure was predicted by homology modelling using the ESyPred3D program [37] using *B. sp* H-257 MGL crystal structure (PDB no. 3RM3) as template (89% identity with EstGtA2). Image of the resulting 3D model was generated using PyMOL and the quality of the model was evaluated using WHAT_CHECK and PROCHECK [38] available in the SWISS-MODEL workspace [39]. Computation of continuum electrostatic and evaluation of salt bridges formation based on the EstGtA2 model was performed using the ESBRI interface [40]. The accessible surface area (ASA) was calculated using the EstGtA2 3D structure model (PDB file) using ASA-View and GetArea (University of Texas, Medical Branch) and the p*K*_a_ values of charged groups involved in putative salt bridges formation was evaluated using the PROPKA server [41].

## Results

### pH-dependent stability of EstGtA2

**Figure 1:**
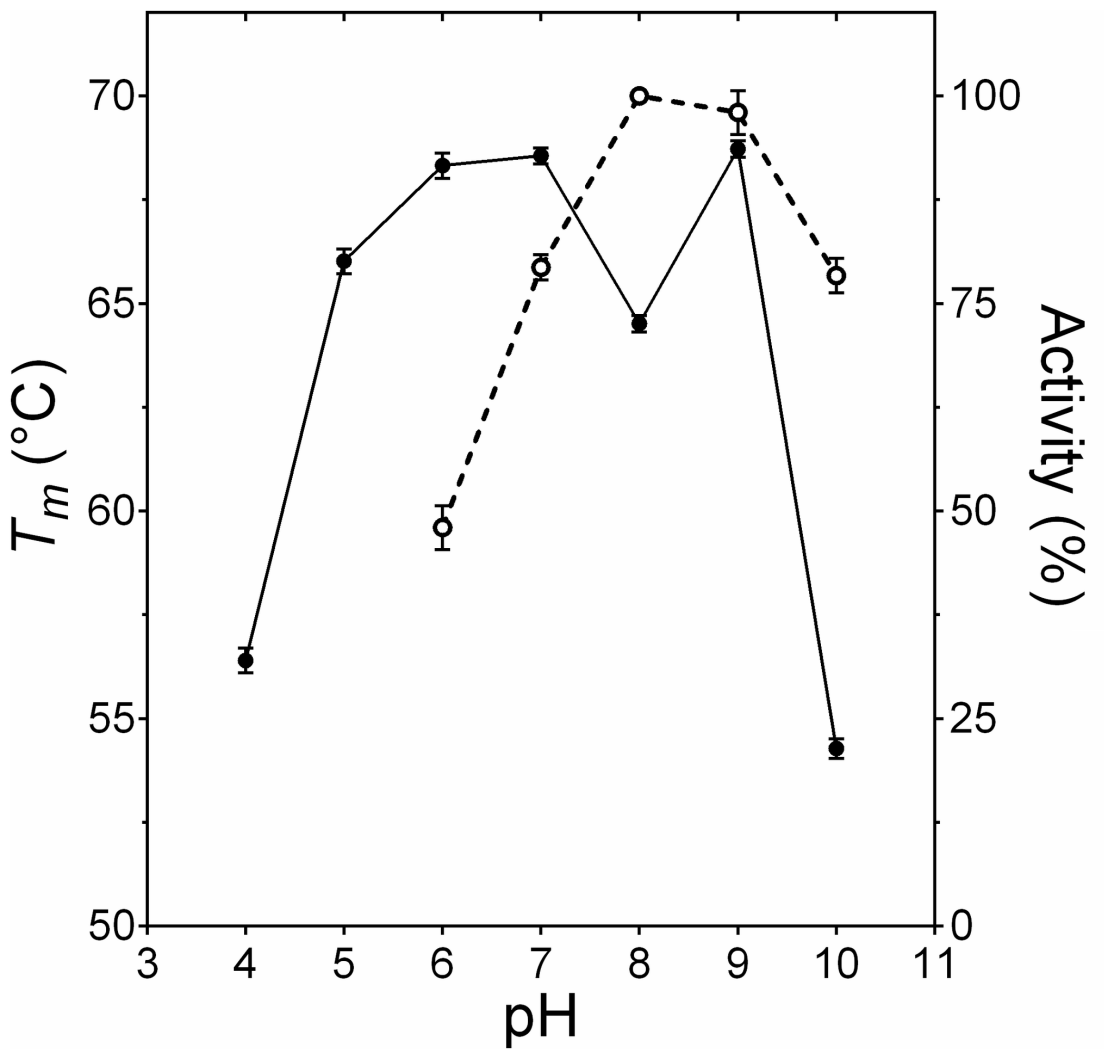
Activity and stability dependence on pH for EstGtA2. The apparent melting temperatures (derived from CD) as function of pH are shown as closed circles. Open circles show relative activity (hydrolysis of *p*NP-octanoate) at different pH values at 50°C (optimal temperature). Standard deviations for *T*_*m*_ ranged between 0.1-0.3°C and do not exceeded 5% for activity. Buffers used were sodium citrate/citric acid (pH4-5), sodium phosphate (pH 6-8), Tris-HCl (pH 9) and CAPS/NaOH (pH 10-11).

EstGtA2 unfolding and refolding was probed as function of pH by monitoring the CD signal at 222 nm as function of temperature. The melting temperatures (*T*_*m*_) for the wild type enzyme ranged from 64 to 69°C between pH 5 and 9. The lowest melting temperatures were recorded for pH 4 and 10 (54 and 55°C respectively, pH limits allowing for folding), Figure 1. Melting temperature decreased slightly at pH 8, which corresponds to optimal pH for activity (at optimal temperature of 50°C). At 25°C, the optimal pH for activity is observed at pH 9-10 and the protein is still active at pH 11, however completely inactivated at pH 12. Partial reversibility of thermal unfolding was only observed under alkaline pHs (pH 7-10). The conformational stability of EstGtA2 calculated from the CD transitions ranged from 8-17 kcal/mol depending on pH. Under acidic condition, aggregation appeared at high temperature leading to irreversible thermal unfolding (data not shown). The high sensitivity to pH suggests that ionized residues and/or salt bridges play a role in EstGtA2 stability.

### Structural model of EstGtA2

**Figure 2:**
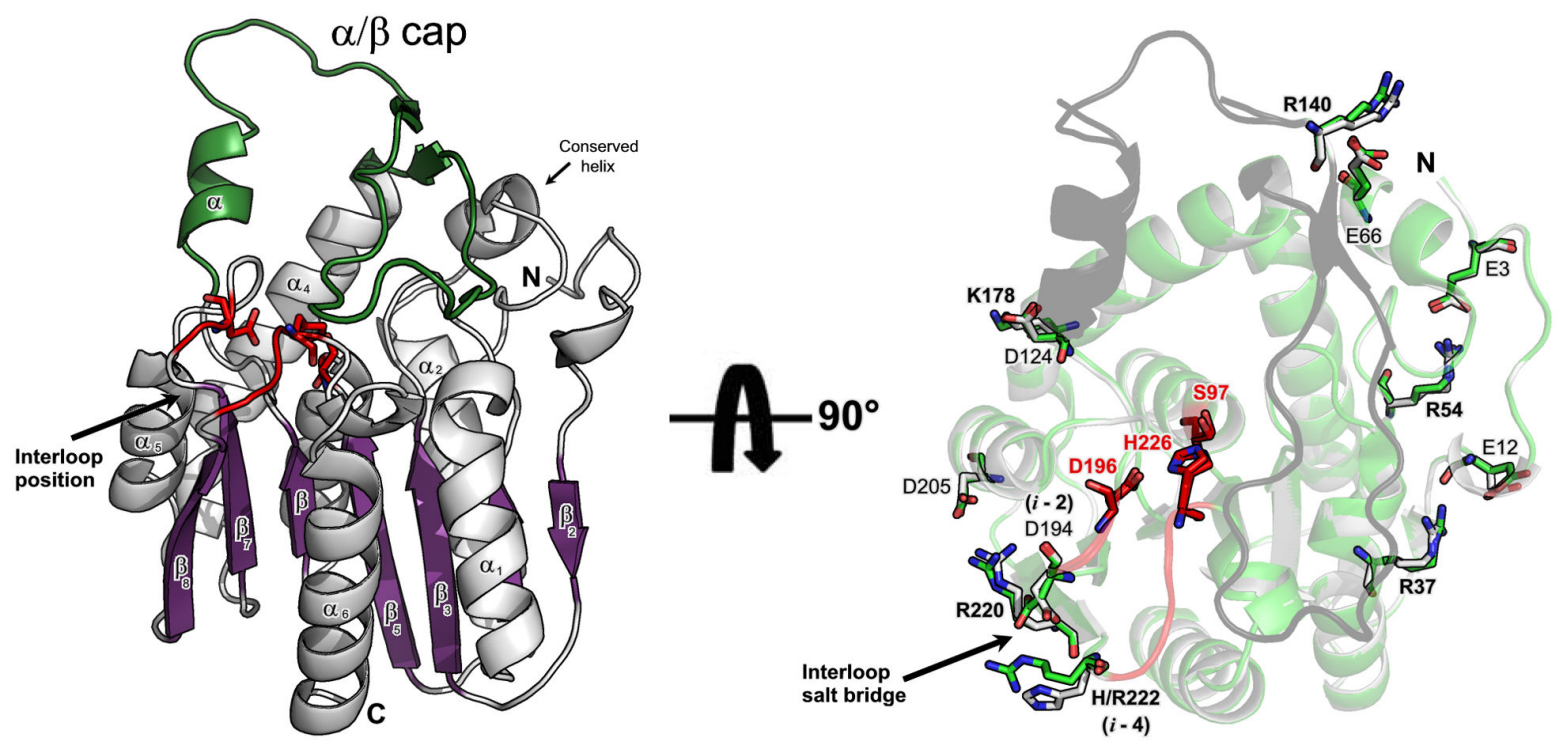
Structure model of EstGtA2. Ribbon structure of EstGtA2 based on the X-ray crystal structure of MGL H-257 (left). The beta sheet is shown in purple and the seven strands are identified as β2-β8, alpha helices are in grey and identified as α1-α6. The cap domain (residues 125-161) is shown in green. The separate conserved helix completing the cap structure is shown. The catalytic triade S97, D196, H226 is shown as red sticks and the interloop salt bridge position (catalytic loops) harbouring the tolerant salt bridge is shown in red. Right panel shows a structural alignment of EstGtA2 model (white) and MGL H-257 crystal structure (3RM3) in light green. The corresponding ribbon structures are almost perfectly superimposed and shown with a 90° rotation view compared to left model. The five salt bridges studied and exclusive to the N’ subfamily are shown (E3-R54, E12-R37, E66-R140, D124-K178 and D205-R220) as sticks. The five basic residues studied by combinatorial mutagenesis are labelled in bold. The interloop salt bridge conserved in (*i* -2, *i* -4) from the catalytic Asp and His residues respectively is identified by an arrow (D194-H222 in EstGtA2) and (D194-R222 for MGL H-257).

In order to identify charged residues likely to impact on stability, the tridimensional structure of EstGtA2 was modeled using the X-ray crystal structure of MGL H-257 (PDB code. 3RM3, 89% residue identity with EstGtA2) [42-44]. As shown in Figure 2, the α/β core comprises a β-sheet formed by 7 β-strands (numbered β2-β8) surrounded by 6 helices (numbered α1-α6). The catalytic triad for EstGtA2 and MGL H-257 is unambiguously identified as Ser97, Asp196 and His226 and the oxyanion hole is formed by Met98 and Phe29. The residues of the binding pocket identified in MGL H-257 are all conserved in EstGtA2 (Figure S1). In addition to the canonical α/β core, a 45 residues cap domain (I125-T164) is found above the main α/β core, a structural feature found in many α/β hydrolases. The cap region is predicted to harbour a small α-helix (A125-A130) and two short anti-parallel β-strands (T141-D143), and (L161-P163) connected by linker regions. A separate α-helix (62-67) interacts with the large cap insertion moiety (I125-T164) forming the cap structure (Figure 2). Comparison of the model with MGL H-257 suggests a high conservation of the residues in the cap region, the binding pocket and the β-sheet. Interestingly, the 27 amino acids that are different in EstGtA2 compared with MGL H-257 are concentrated in α-helices and loops of the core of EstGtA2 (Figure S2). These mutations may contribute to the flexibility of the binding pocket or the catalytic cleft, which in turn would explain the difference of specificity between EstGtA2 and MGL H-257 observed (EstGtA2 is able to hydrolyze tributyrin while MGL H-257 cannot) [43]. The Ramachandran plot and the model refinement statistics are presented in supporting information (Figure S3). The conservation of 89% of residues between EstGtA2 and MGL H-257, and the quality assessment of the model, supports the hypothesis that EstGtA2 and MGL H-257 adopt a very similar structure.

### Multiple conserved salt bridges in EstGtA2

**Figure 3:**
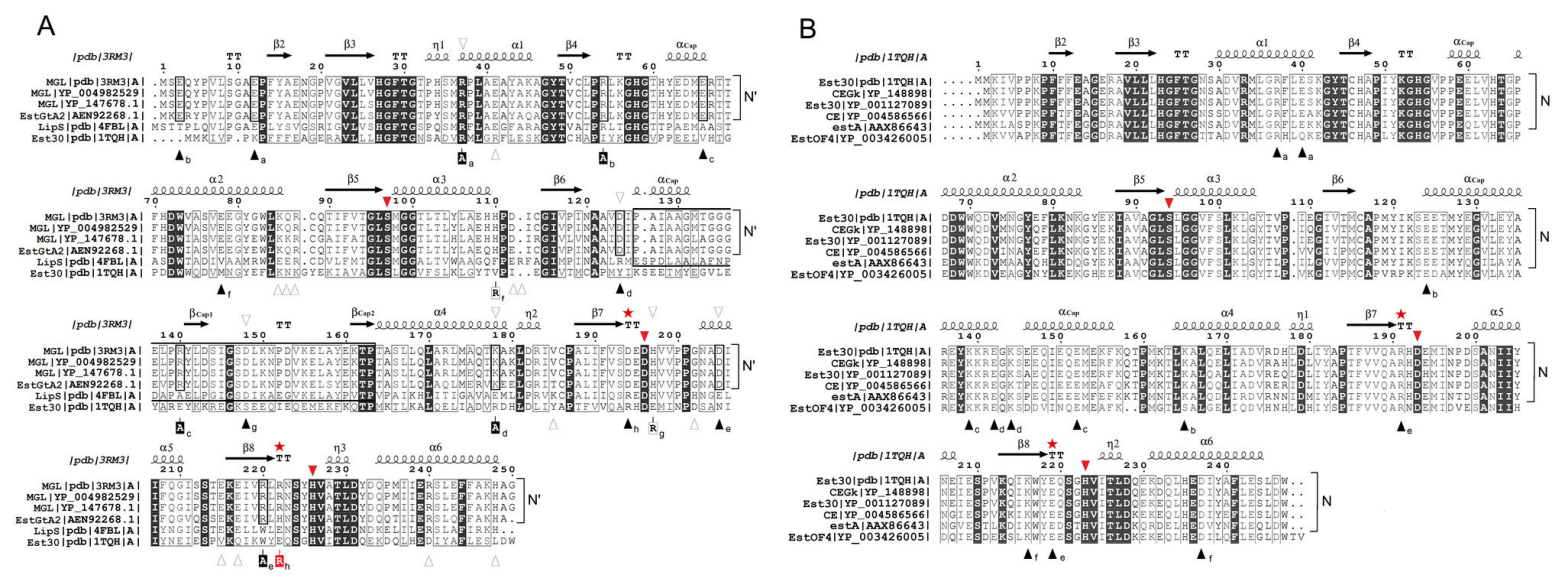
Distinctive salt bridge patterns conserved within family XV and XIII. Panel A: Multiple sequence alignment for N’ subfamily enzymes (MGL from *Bacillus* sp. H-257, *G*. *thermoleovorans*, *G*. *kaustophilus* and EstGtA2 from *G*. *thermodenitrificans*) and representative of the LipS (4FBL) and N subfamily (1TQH). The secondary structure elements from MGL H-257 (3RM3) are shown on top. The cap domain is boxed (thick line). Residue numbering is based on EstGtA2 and MGL H-257. The residues of the catalytic triad are identified by a triangle above the MSA. The seven N’ conserved salt bridges: E3-R54, E12-R37, E66-R140, H110-E78, D124-K178, H197-D148 and D205-R220 are identified by close triangles below the MSA with same letter. Residues shaved by alanine-scanning are identified by a black box below the MSA. Open triangles refer to LipS salt bridges pattern. The tolerant interloop salt bridge located in (*i* -2, *i* -4) from the catalytic Asp and His respectively is identified with a red star above the MSA. The alanine or arginine substitutions are indicated by a box A or R. Panel B: Multiple sequences alignment of N-related subfamily enzymes, compared to LipS and MGL (from N’ subfamily). Six exclusive salt bridges are identified by arrows: R37-E40, E124-K165, K139-E152, E142-K144, R191-E219 and K216-D237. Numbering is based on Est30 (1TQH).

Conserved salt bridges were predicted in the structures of EstGtA2 and MGL H-257 using ESBRI with a cut-off distance of 4 Å. In addition, the crystal structure for MGL H-257 were solved from a protein crystal obtained at pH 7.5, which is very close to the pH value were these salt bridges were studied in EstGtA2 (pH 8). Eight salt bridges were suggested in EstGtA2 and MGL H-257: E3-R54 (3.3 Å), E12-R37 (3.9 Å), E78-H110, E66-R140 (3.6 Å), D124-K178 (3.3 Å), D148-H197, D194-R/H222 (4.0 Å) and D205-R220 (3.6 Å). Seven out of those eight salt bridges are identical in EstGtA2 and MGL H-257. In the eighth, an arginine in MGL replaces the histidine found in EstGtA2 at position 222 (D194-H222 in EstGtA2; D194-R222 in MGL 257). This salt bridge links the loop between strand β7 and helix α5 (displaying the catalytic Asp residue) to the loop between strand β8 and helix α6 (harbouring the catalytic histidine, see Figures 2 and 3) and is conserved at positions *i* -2 and *i* -4 from the respective catalytic residues.

The calculated solvent accessibility for the salt bridges-forming charged residues predicts that most of these salt bridges are relatively buried except for the interloop salt bridge which is highly exposed at the protein surface (Table S2 in File S1). The E3-R54 salt bridge is the most buried ion pair; as such one might expect a more important contribution to stability from this electrostatic interaction. The theoretical prediction of the p*K*_a_ values for the residues involved in salt bridges range from 3.78 to 4.2 for glutamates and from 3.56 to 3.74 for aspartates. The p*K*_a_ values for arginine and lysisne residues ranged from 11.09 to 13.85 while for histidines they varied between 6.03 and 6.64. It should be noted that the three salt bridges involving histidine in EstGtA2 are not considered for these predictions, since they are not expected to be formed in the pH range (pH 8-10) studied here.

**Figure 4:**
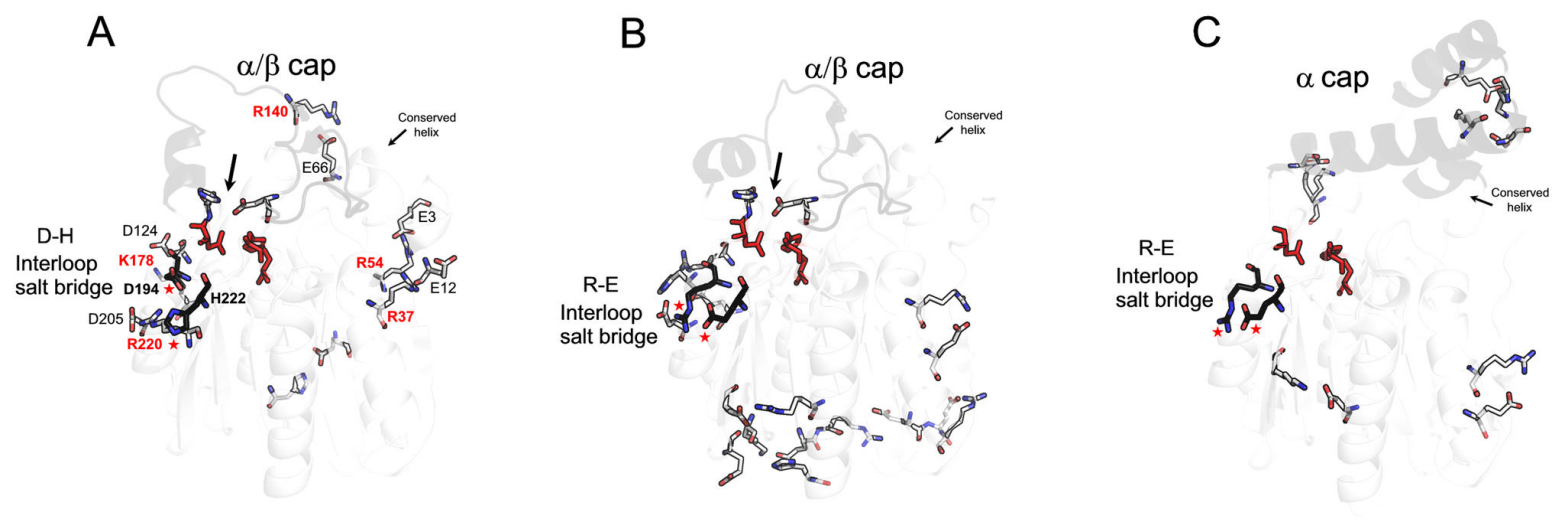
Distinctive salt bridge patterns conserved in related lipolytic enzymes. Structural comparison of the salt bridges content for EstGtA2 (A) LipS (4FBL) (B) and Est30 (1TQH) (C) is shown. At variance with the conserved interloop bridge, the 5 selected salt bridges in EstGtA2 are absent in LipS- and N-related enzymes. Residues exclusive to the N’ subfamily and studied by alanine-scanning mutagenesis are shown in red. Structure of the cap domain is shown in dark gray. The conserved helix of the cap between the three structures is identified. LipS structure share features from N’ and from N subfamilies. The arrow shows salt bridge conserved between EstGtA2 and LipS. The residues forming the interloop salt bridge are shown and identified on the respective structures.

Salt bridges from closely related enzymes were also examined. Salt bridge composition for EstGtA2 (N’ subfamily), LipS and Est30 (N subfamily) is shown in Figure 4 and Table S3 in File S1. Three distinctive patterns are observed, wherein salt bridge-forming residues show different separations in sequence and in space. These different patterns are each conserved within closely-related homologs. Five bridges were exclusively conserved in lipolytic enzymes from the N’ subfamily: E3-R54, E12-R37, E66-R140, D124-K178 and D205-R220, but absent from the recently characterised LipS (PDB code. 4FBL) or from the closely related N subfamily Est30 (PDB code. 1TQH) as shown in Figures 3 and 4. An additional multiple sequence alignment was built for N subfamily representatives (Figure 3, panel B) and revealed a series of 6 bridges found exclusively in N subfamily. Such exclusive bridge composition appears to be a hallmark for quick recognition of N’- or N-related enzymes from genomic analysis.

### Salt bridges as hallmarks of the N’ subfamily

The first salt bridge among the five bridges conserved in the N’ subfamily, E3-R54, links the N-terminal end of EstGtA2 to the loop after strand β3. Three hydrogen bonds and electrostatic interactions between the guanidinium group of R54 and the carboxylate of E3 side-chain are predicted involving the resonance form and the positive charge delocalisation of the guanidinium group of R54 (Figure S4). Two additional hydrogen bonds are formed, one between the R54 guanidinium and the backbone carbonyl oxygen of H58 and one between the NH group of the R54 backbone and the OH group of the S76 side chain. In addition, the aliphatic moiety of R54 side-chain is buried in a hydrophobic cluster with two proline residues, (P13 and P33). The E12-R37 salt bridge also functions to link the N-terminal end of EstGtA2 to the core through a hydrogen bond found between the guanidinium group of R37 side-chain and the carboxylate of E12 side-chain.

Another noteworthy predicted salt bridge between E66-R140 links the large insertion of the cap domain to the separated helix of the cap formed by residues 61 to 67. In addition to salt bridge between the guanidinium of R140 and E66 carboxylate, residue R140 form an additional hydrogen bond with the OH group of Y141. The salt bridge formed between D124 and K178 is the second most buried ion pair. A hydrogen bond is formed between the ε-amino group (NH3^+^) group of K178 and the carboxylate group of D124. Two additional hydrogen bonds are formed between D124 and N203, one between the backbone carbonyl oxygen of D124 and the NH_2_ side-chain amide group of N203 and one between the backbone NH group of D124 and the oxygen of the side-chain amide group of N203. The aliphatic moiety of K178 side-chain also interacts with the V123 side-chain. The salt bridge D205-R220 contains only one hydrogen bond and electrostatic interaction between side-chains and links the strand β8 to the helix α5 (Figure S5). The five exclusive salt brides displayed important interactions and might play a role in EstGtA2 stability.

**Figure 5:**
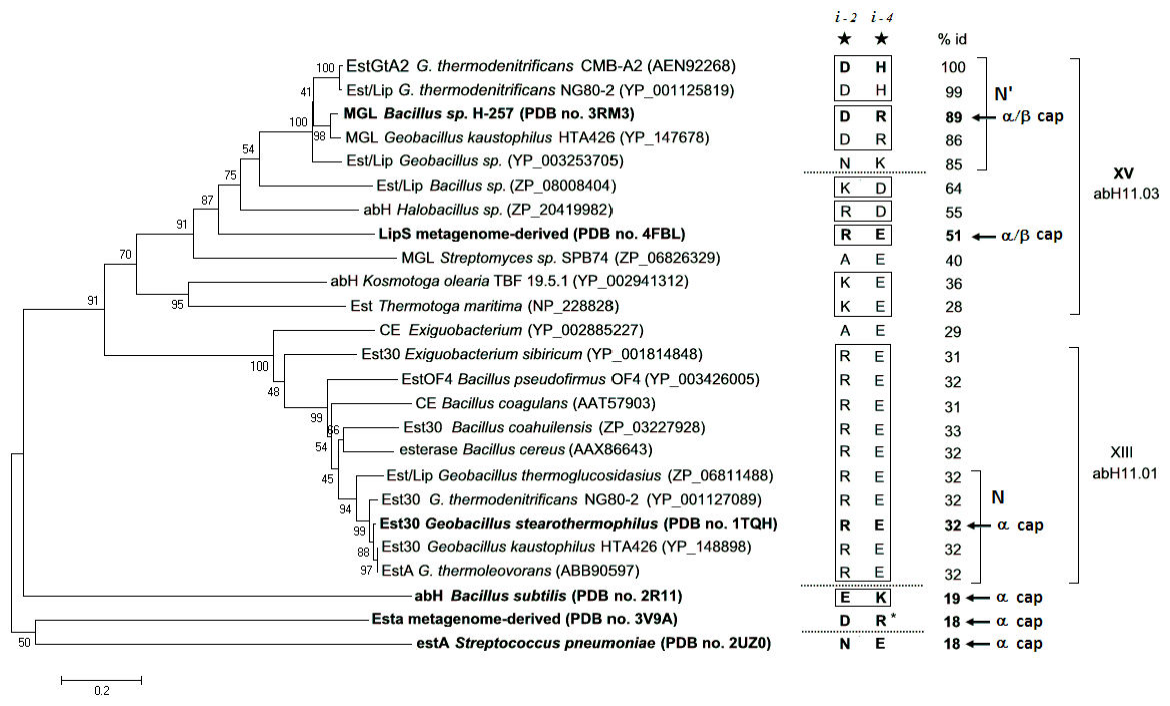
Evolution of the interloop salt bridge near the active site. A phylogenetic analysis of bacterial lipolytic enzymes related to family XV and XIII on the separation of the N’ and N subfamilies. Sequences with solved crystal structures displaying the interloop salt bridge located in (*i* -2, *i* -4) from the catalytic Asp and His respectively are shown in bold. The corresponding ion pairs are shown right to the tree and annotated with a star and position relative to catalytic residues. Numbers show percentage identity compared with EstGtA2. Dashed lines indicate polarity reversals observed at the conserved interloop salt bridge position. The cap structure is indicated for X-ray resolved lipolytic enzymes. Sequences were assigned to N,N’and abH11 families (taking into account enzymes classification in Lipase Engineering Database). The phylogenetic and molecular evolutionary analyses were conducted using MEGA version 5. The evolutionary history was inferred using the neighbor-joining method and the evolutionary distances were computed using the Poisson correction and are in the same units of the number of amino acid substitutions per site.

Of the eight salt bridges found in EstGtA2 and MGL H-257, only two are conserved in the homolog LipS. The first one is D148-H197 (EstGtA2), corresponding to D179-H228 (in LipS), which links the cap domain to the α/β core. The second conserved salt bridge is located at the interloop position. Despite the low conservation of most salt bridges in EstGtA2 compared to enzymes from the closely related N subfamily (family XIII) or recently identified LipS [8], a particular interloop salt bridge appears to be conserved across most members of these recently identified families, but with high tolerance to mutations. A phylogenetic tree (Figure 5) shows the evolution and polarity reversal at this conserved salt bridge position within N and N’ subfamilies, and for other distant bacterial α/β hydrolases. This salt bridge is formed between R225 and E253 in LipS, R191 and E219 in Est30, D194 and R222 in MGL 257 and D194 and H222 in EstGtA2 (Figures 3 and 4), to name a few. The polarity of the bridge is switched from acid-base polarity in the N’ subfamily, to base-acid polarity in N subfamily (Figure 5). This salt bridge tethers loops directly connected to active site segments, and is conserved at residue positions *i*- 2 and *i* -4 from the catalytic Asp and His respectively. The bridge is not perfectly conserved however (see for example YP_003253705 and YP_002885227 in Figure 5), suggesting that its presence is not absolutely necessary. This bridge was studied here considering its proximity to active site residues, and possible impact on catalytic properties. Given that histidine is the positive partner at this position in EstGtA2, we investigated a possible impact of replacing His by Arg on pH-dependent properties.

### Combinatorial alanine-shaving of salt bridge-forming residues

**Figure 6:**
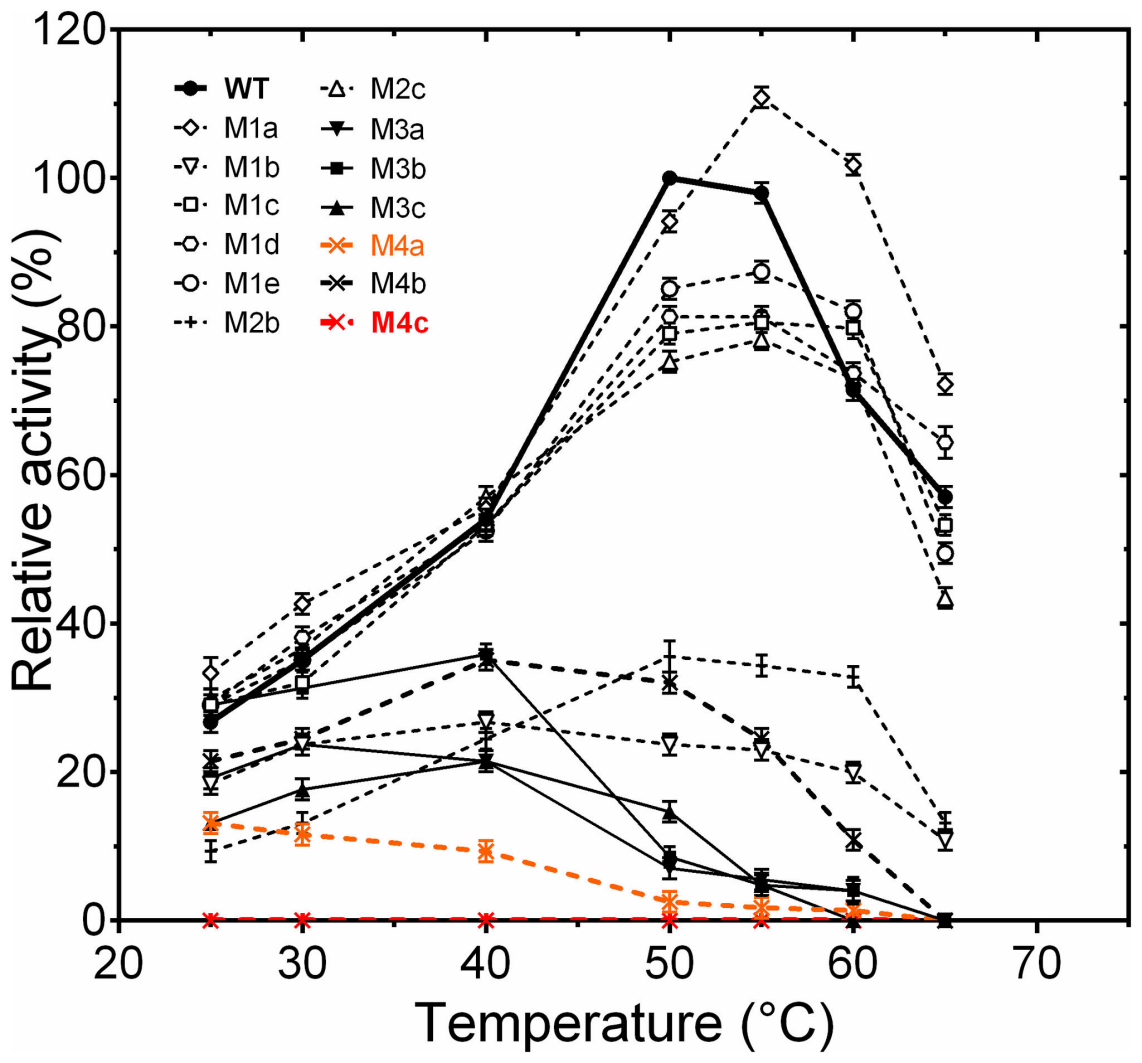
Activity profile for wild type and mutants. Specific activity (µmol min^-1^ mg^-1^) was measured from 25 to 65°C for the wild type and mutants in 20 mM sodium phosphate pH 8 using *p*NP-octanoate as substrate. The initial rates (V_0_) were measured below substrate saturation and than reflect *K*_cat_/*K*_m_ conditions. Each point (specific activity values) was obtained from three different experiments and reported relative to wild type at 50°C (optimal conditions). Standard deviations do not exceed 5%. The wild type is shown as close circles and bold line. The curve for the quadruple mutant M4a is shown in orange and in red for M4c. The mutation R37A enhances the activity and shift the optimal temperature compared to wild type, the relative activity at 25 versus 60°C are listed in Table 1.

**Figure 7:**
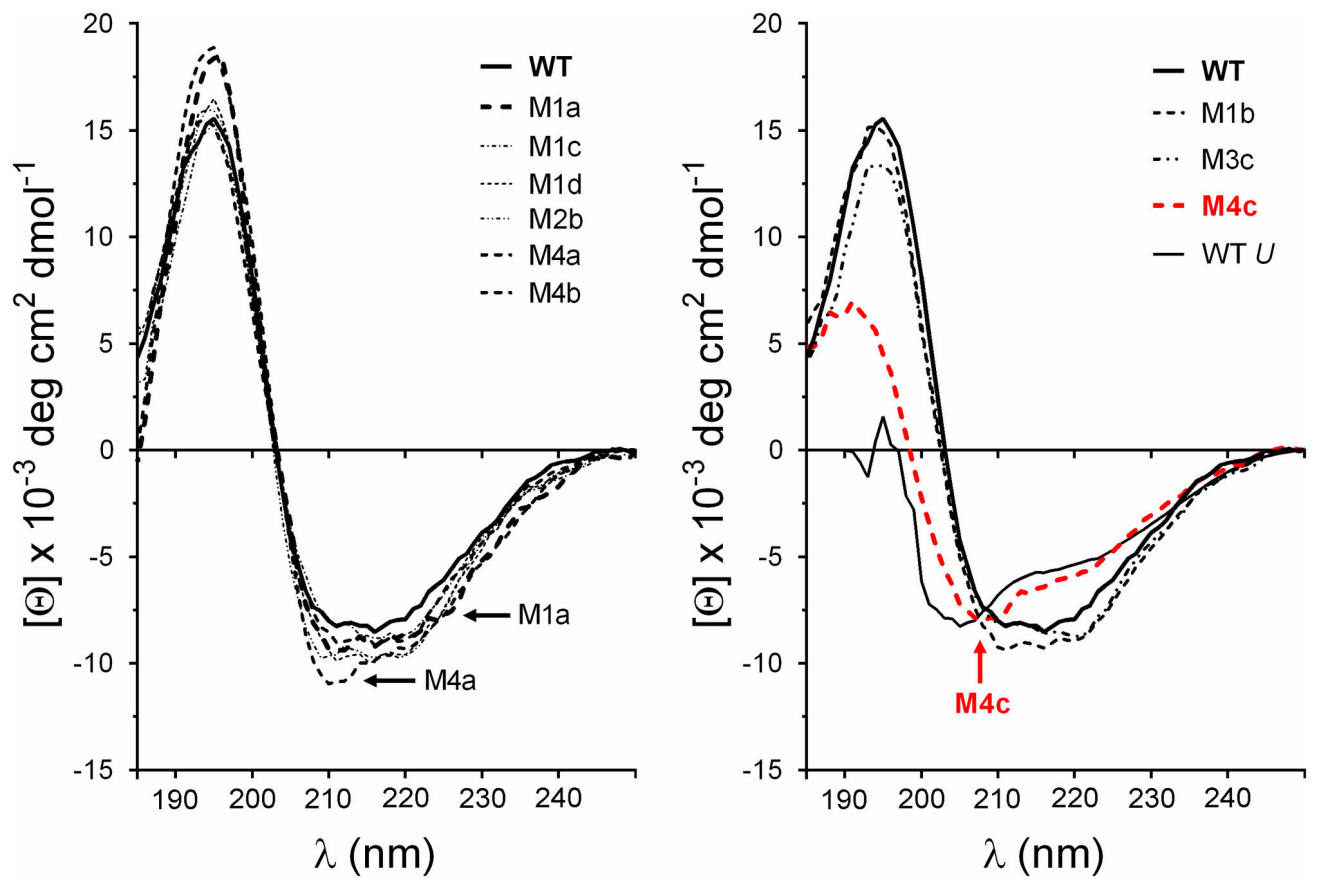
Conformational analysis by far-UV circular dichroism. Far-UV CD spectra were recorded at 25°C with 0.5 mg/ml of protein in 20 mM sodium phosphate pH 8. The wild type is shown in thick black lines. Left panel shows that most mutants are folded. Right panel shows misfolding of M4c. The quadruple mutant M4c spectra (red curves) were similar to spectra of unfolded EstGtA2 (WT *U*), wild type spectra recorded at 90°C.

**Figure 8:**
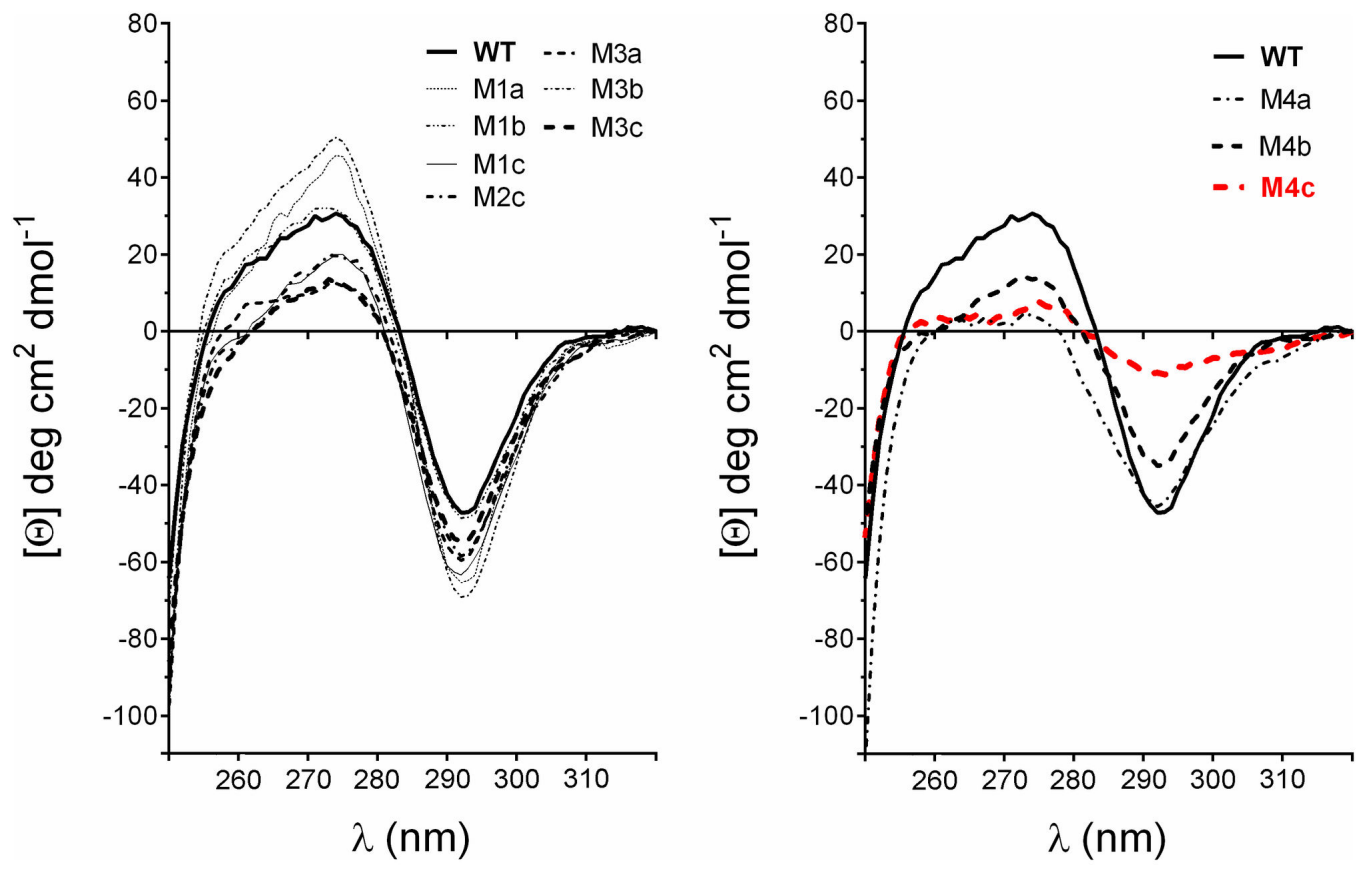
Conformational analysis by near-UV circular dichroism. Near-UV CD spectra were recorded at 25°C with 0.5 mg/ml of protein in 20 mM sodium phosphate pH 8. While all mutants had a strong minimum at 292 nm, M4c (red curve) had a weak signal throughout the range studied, suggesting loss of packing near aromatic residues.

Here the contribution to stability of the five salt bridges identified above (exclusively conserved in N’ family) was studied further using combinatorial alanine-scanning mutagenesis. To this end, the bridges containing Arg or Lys residues as the positively charged partner (salt bridges E3-R54, E12-R37, E66-R140, D124-K178 and D205-R220), were progressively disrupted by substituting alanine for K or R, Figure 2, Table 1. Single, double, triple and quadruple mutants were produced as depicted in Table 1. All mutants were active at room temperature (Figure 6) and correctly folded as suggested by near- and far-UV CD, except for the quadruple mutant M4c spectra (Figures 7 and 8). These mutations resulted in complete inactivation of the enzyme. All single mutations caused an increase in the activity at room temperature and moderate temperature (below 50°C, the optimum temperature for the wild type), perhaps due to an increased flexibility of the core compared to EstGtA2 at this temperature (see Figure 6 and Table 1). The increased activity observed for single mutants was also observed at 60°C and on *p*NP ester of longer acyl chain-length (*p*NP-C12). This appears to be due to a general improvement of activity rather than a shift in chain-length specificity (data not shown). The mutation R37A is the most activating one with a 1.2 and 1.5 fold increase activity at 25 and 60°C. At 60°C, the R54A mutation is the most destabilizing single mutation, as mentioned above, this bridge is relatively buried and involved in a network of interactions (Figure S4). Triple mutants were nearly as active as the wild type enzyme at 25°C, but were inactivated at 60°C where the wild type EstGtA2 retained more than 70% activity. Among the three quadruple mutants analysed, M4b was as active as EstGtA2 at 25°C and at variance with triple mutants, it remains active at 60°C (40% of wild type). M4a lost 70% of activity at 25°C, and became inactive at 60°C. The particular set of mutations leading to M4c led to total inactivation of this mutant (Table 1). In general, the R37 mutation appears to stimulate (or prevent inactivation) activity (in M1a, M4b), while R54 is present in less active mutants (in M1b, M3a, M3b and M4c).

**Table 1:** Impact of salt bridges disruption on EstGtA2 activity and stability.

Protein	Mutations	*T*_*m*_ (°C)	Δ*T*_*m*_ (°C)	Activity (%)^a^	Activity (%)^b^
WT	-	64.2 ± 0.11	-	100	100
M1a	R37A	58.6 ± 0.31	-5.6 ± 0.21	123 ± 3.6	143 ± 3.7
M1b	R54A	55.6 ± 0.25	-8.6 ± 0.18	68.1 ± 2.3	27.2 ± 5.1
M1c	R140A	56.6 ± 0.23	-7.6 ± 0.17	108 ± 5.1	112 ± 2.1
M1d	K178A	55.3 ± 0.23	-8.9 ± 0.17	108 ± 2.4	103 ± 2.3
M1e	R220A	55.4 ± 0.15	-8.8 ± 0.08	108 ± 0.8	68 ± 0.9
M2a	R37A/R220A	55.6 ± 0.18	-8.6 ± 0.15	n.d.	n.d.
M2b	R140A/R220A	54.1 ± 0.19	-10.1 ± 0.15	60.2 ± 4.5	92.1 ± 3.6
M2c	K178A/R220A	53.6 ± 0.29	-13.6 ± 0.20	112 ± 3.3	76.2 ± 4.1
M3a	R54A/R140A/R220A	53.9 ± 0.31	-10.3 ± 0.22	71.3 ± 2.7	4.56 ± 2.1
M3b	R54A/K178A/R220A	52.3 ± 0.17	-11.9 ± 0.14	108 ± 2.7	8.31 ± 1.7
M3c	R140A/K178A/R220A	50.4 ± 0.10	-13.8 ± 0.09	47.3 ± 3.3	0
M4a	R37A/R54A/R140A/R220A	53.2 ± 0.23	-11.0 ± 0.17	47.2 ± 2.8	0
M4b	R37A/R140A/K178A/R220A	49.9 ± 0.30	-14.3 ± 0.21	79.1 ± 1.9	14.5 ± 2.3
M4c^U^	R54A/R140A/K178A/R220A	-	-	0	0
WT^c^	-	55.1 ± 0.21	-	100	100
ISB^c^	H222R	57.6 ± 0.18	+ 2.5 ± 0.24	98.2 ± 0.8	254 ± 5.1

Melting temperatures and specific activities were measured at pH 8. The amount of pNp-octanoate hydrolyzed as a function of time. Specific activities (µmol min^-1^ mg^-1^) are reported in percentage (relative to the wild type in respective temperature). The initial rates (V_0_) were measured under saturation and than reflect *K*_cat_/*K*_m_. Each thermal denaturation curves or rate of hydrolysis were collected at least three times. The *T*_*m*_ and activity values are average of three different experiments. Standard deviations based on respective triplicates are shown.

*U*. Unfolded and completely inactivated at 25°C.

a Activity relative to wild type at 25°C

b Activity relative to wild type at 60°C

c Proteins properties recorded at pH 10 using 20 mM CAPS buffer.

**Figure 9:**
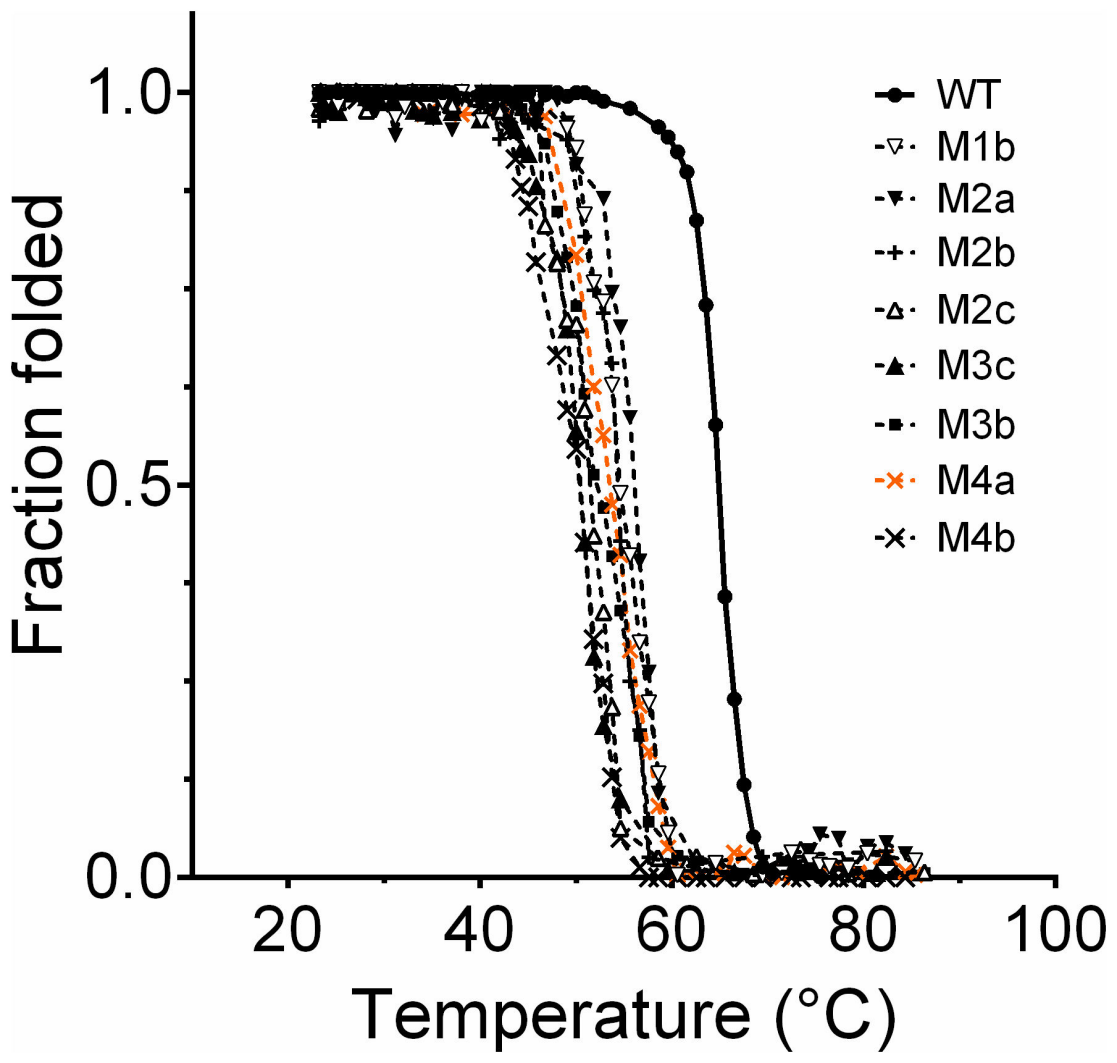
Thermal unfolding of EstGtA2 and mutants. Thermal unfolding curves recorded from 25 to 90°C were expressed as fraction folded derived from the CD signal at 222 nm as function of temperature. Samples were made of 0.5 mg/ml of protein in 20 mM sodium phosphate pH 8. EstGtA2 had the highest denaturation temperature among all versions studied. The quadruple mutant M4b is the least stable. The quadruple mutant M4a is shown in orange.

Thermal denaturation curves were measured by monitoring the molar ellipticity at 222 nm as function of temperature and are expressed as fraction folded (Figure 9). The melting temperatures (*T*_*m*_) were calculated by plotting Δ*G* as function of temperature, where *T*_*m*_ = Tat Δ*G* = 0 (Figure S6). The thermodynamic parameters and activity measured for the wild type and mutants are shown in Table 1.

**Figure 10:**
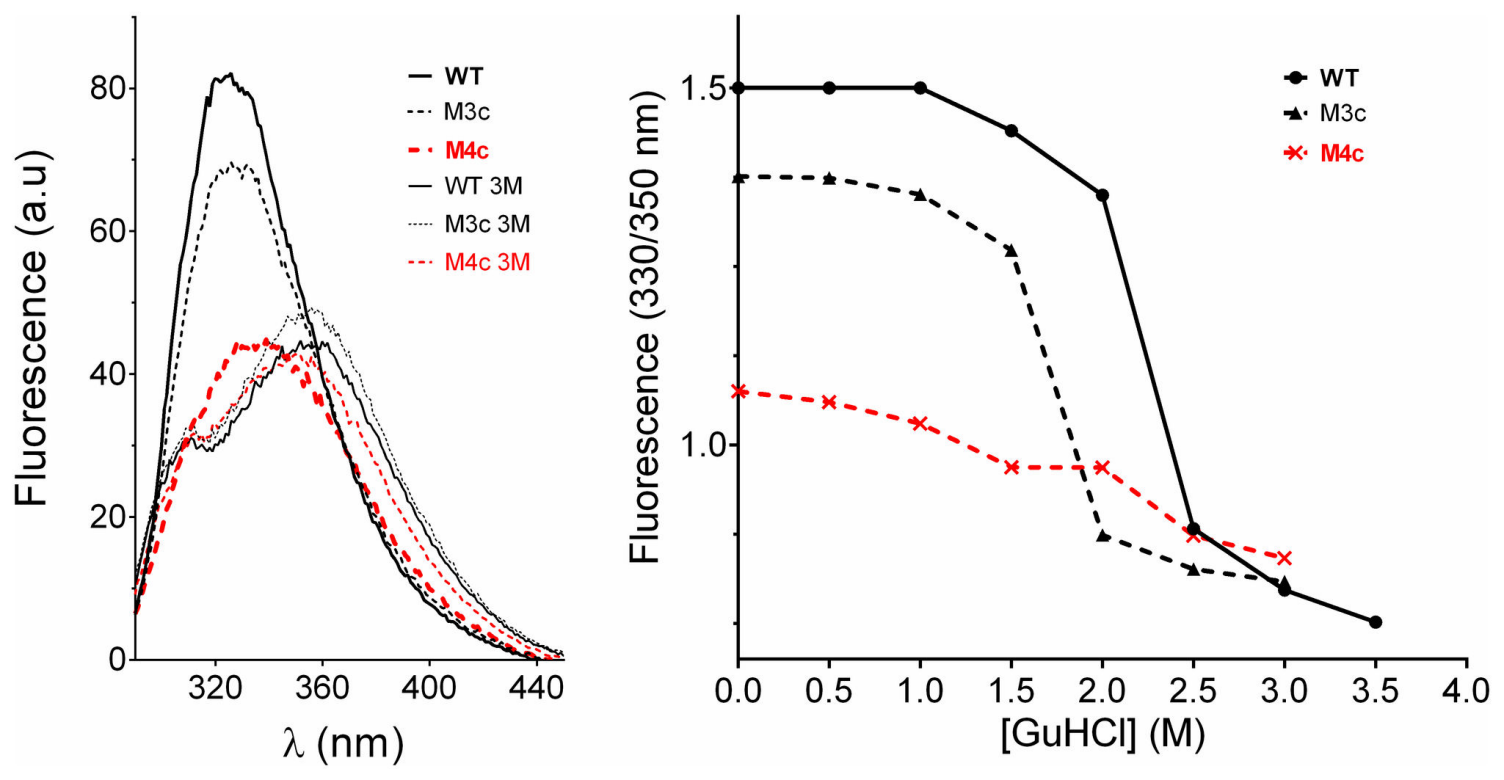
Intrinsic fluorescence. Panel A: Fluorescence spectra recorded for WT, M3c and M4c (red curves) at 25°C with 0.1 mg/ml at pH 8 in the same buffer (top spectra), and with 3 M GuHCl (bottom spectra). Panel B: Corresponding chemical denaturation curves are shown. Fluorescence peak was shifted toward longer wavelength upon denaturation, indicating hydration of aromatics. M4c showed hydration of aromatics in the absence of denaturant.

Despite the fact that all mutants (except M4c) folded correctly and retained activity at low temperature, all the mutations were significantly destabilizing for EstGtA2. As shown in Figure 10 and Table 1, the R37A, R54A, R140A, K178A and R220A single mutants were characterised by lower *T*_*m*_ (with Δ*T*_*m*_ ranging from -5.6 to 9.8°C). The double mutants had a decrease in *T*_*m*_ ranging from -8.6 to -13.6°C. Triple mutants were even less stable than the single and double mutants, with drops in *T*_*m*_ ranging from -10.3 to -13.8°C. The worst scenarios were observed with quadruple mutants, where decrease in *T*_*m*_ ranged from 11 to 14.3°C. Only single mutants retained activity above 60°C (see Figure 6). Interestingly, the triple mutant R140A/K178A/R220A (M3c) showed a reduced *T*_*m*_ of 11.8°C but folded correctly and was active at room temperature (same observation for the two other triple mutants). However, they were much less stable and were inactivated at 60°C. Interestingly, when the R54A mutation was added to the three mutations of M3c to form the quadruple mutant R54A/R140A/K178A/R220A (M4c), the folding of the enzyme was compromised. The quadruple mutant M4a shows considerable conformational changes and was almost inactivated at room temperature, being completely inactivated at 50°C. The quadruple mutant M4b was much more active than M4a (almost equal to the wild type at 25°C) but was inactivated over 60°C (Figure 6). Interestingly the mutant M4b contains the mutation R37A which may play a compensatory role, while M4a contains R54A which seems to be the most destabilizing single mutation. When R54A is combined with R140A/K178A/R220A, protein folding is prevented. The double mutant M2c showed a reduction in *T*_*m*_ that is comparable to triple mutant M3c as well as M4b. It appears that mutation R140A which decreased *T*_*m*_ by 9.3°C in the single mutant M1c did not destabilize further the M3c or M4b derivatives, or that some compensatory mechanisms are at play in multiple mutants.

### Misfolding of M4c

As shown in Figures 7 and 8, important modifications in the far- and near-UV CD spectra were observed for the quadruple mutant (M4c) compared to wild type and the other mutants. The molar ellipticity in the far-UV region shifted from the typical spectral shape observed for α/β structures to a spectrum having the hallmarks of protein denaturation (negative peak at 203 nm, loss of signal in the range of 210-230 nm). Calculation of secondary structures for the native wild type enzyme predicted 35% α-helices, 20% β-sheets, 20% turns and 25% unordered structures. These values are close to those calculated with the atomic model of MGL 257 3RM3 (35% helices, 16% strands). The quadruple mutant calculations revealed a major reduction of α-helices (-23%) with a concomitant gain in beta and unordered structures (+7%) compared to wild type. The near-UV CD spectra that result from compactness in the surrounding of aromatic residues changed slightly for M3a, M3b, M3c, M4a, and M4b. This suggests some modification in the packing, a possible consequence of the change in bulk at the mutated position (R being much larger than A). This effect seemed to be more drastic for M4c which showed a major decreased in CD signal intensity at 290 nm (possibly corresponding to tryptophan). This observation suggests an increased mobility of the tryptophan side-chains possibly as a result of the unfolded state of this mutant.

The fluorescence spectra (Figure 10) also suggest misfolding of M4c as observed by CD. An observed shift of the fluorescence emission peak (λ_max_) from 320 to 355 nm indicates that hydration occurred in the hydrophobic core of the protein and/or that polar contacts arose in the immediate environment of the originally highly buried tryptophan residues Trp73 (0) and Trp83 (0.19) (where numbers in parentheses indicate solvent accessibility calculated in the folded structure). Chemical denaturation experiments were performed as shown in Figure 10, panel B. The wild type EstGtA2 shows a [GuHCl]_½_ = 2.2 M in comparison with 1.75M for M3c. The quadruple mutant M4c did not show any transition or shift in λ_max_ indicating that tryptophan residues are already exposed to solvent in the buffer (in the absence of denaturant). Such results confirm that M4c is unfolded and consequently inactive.

### Activation and specificity modification by R37A

Although the R37A mutation is destabilizing (lowering the *T*_*m*_), it increased EstGtA2 activity at low temperature and shifted the optimal temperature from 50 to 55°C. The activation effect seems to be coupled with a change in specificity. The wild type EstGtA2 hydrolyzes tributyrin (C4) but cannot hydrolyze long-chain triglycerides (TAG) such as olive oil (C16-18). Surprisingly, the mutants M1a (R37A) showed activity on emulsified olive oil-agar plates suggesting hydrolysis of long-chain triglycerides. Three more mutants M2a, M4a and M4b also showed hydrolysis of olive oil on emulsified agar plates. Interestingly these mutants all contain the R37A mutation (Figure S7). Every mutant that contains the R37A mutation shows the emergence of this new activity suggesting that mutation at position 37 is essential for this new catalytic ability. A similar conclusion was drawn for the rumen extracted esterase R.34 in which the formation of a single salt bridge turn the enzyme into a true lipase without modification of the shape size or hydrophobicity of the substrate-binding pocket that are usually considered to be essential for chain-length specificity [23]. The exact mechanism by which removing of the arginine residue at position 37 lead to the emergence of this new function in EstGtA2 is under investigation.

### Modification of the (*i* -2, *i* -4) interloop salt bridge

**Figure 11:**
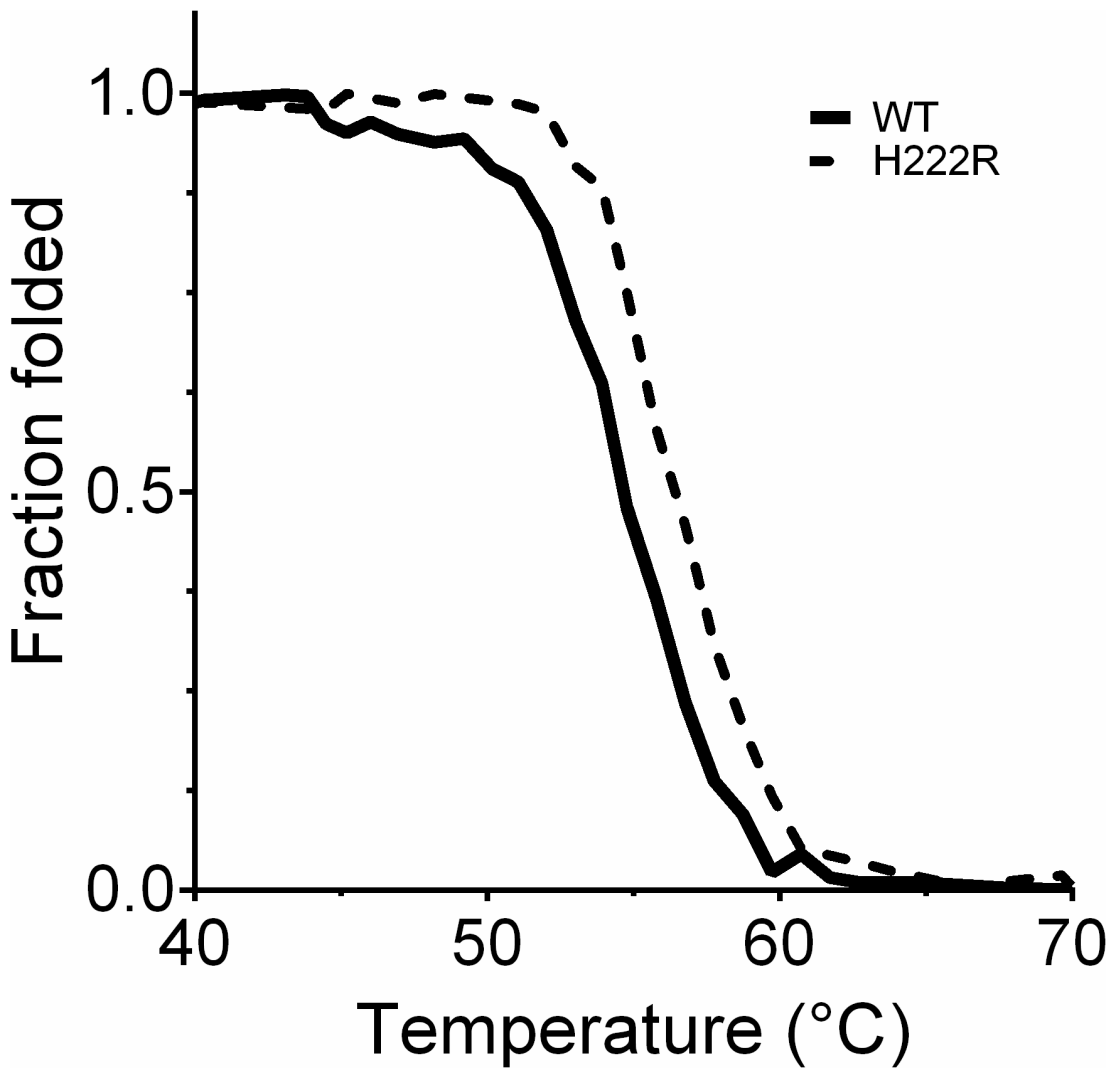
Modification of the interloop salt bridge in EstGtA2. The conserved inter-loop salt bridge found in EstGtA2 (D194-H222) was modified to resemble that of 3RM3 (D194-R222). Figure shows thermal unfolding curves calculated from the CD signal at 222 nm as function of temperature for the wild type enzyme and for the H222R mutant (protein concentration of 0.5 mg/ml in 20 mM CAPS pH 10). The mutation of H for R in position 222 led to increase the denaturation temperature by 2.1°C.

**Figure 12:**
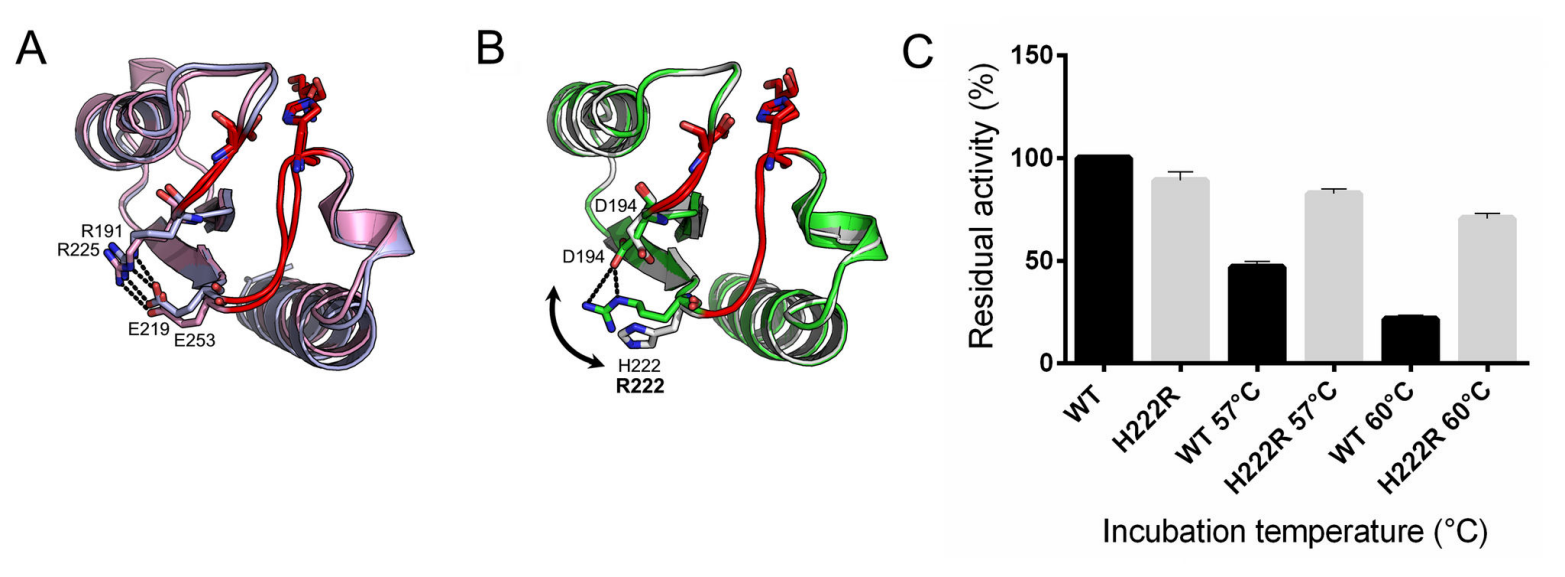
Impact of the new interloop salt bridge on EstGtA2 thermostability under alkaline pH. A. Structural alignment of the interloop portion for Est30 (blue) and LipS (pink). B. Structural alignment for MGL-H257 (green) and EstGtA2 (white), salt bridge at the conserved interloop position is shown for respective structures. Arrow shows change reversal for MGL-H257 and EstGtA2 (N’ enzymes). The proteins (wild type or H222R mutant) were incubated for 10 min at the indicated temperature and the residual activity on *p*NP-octanoate (compared with non-incubated enzymes) was measured at 25°C in 20 mM CAPS buffer pH 10.

A highly conserved interloop salt bridge was identified in distantly related bacterial lipolytic enzymes as discussed above. The bridge was modulated by replacing His222 by Arg which turns the bridge to Asp194-Arg222, a pairing similar to the one found in MGL H-257 and in MGL from *G. kaustophilus* from the N’ subfamily (Figure 5), so this mutation should be easily accommodated. The effect of this substitution on thermal stability was investigated further using CD spectroscopy. The pH-dependent thermal unfolding profile of the wild type and of the H222R mutant was recorded from pH 4 to 10. Results showed that the H222R mutation contributed to increase the apparent *T*_*m*_ value under alkaline pH (at pH 10) by +2.1°C (Figure 11). Activity measurements confirmed that H222R was active over the same pH range as the wild type EstGtA2 (pH 4 to 10) with optimal activity at pH 8 (data not shown). However, at pH 10, the H222R mutant retained more residual activity at room temperature after heating, with 89% left compared to 46% for the wild type enzyme (after heating at 57°C) and 74% left compared to 22% for the wild type after heating at 60°C (Figure 12). These results can be explained by the difference in p*K*_*a*_ between the histidine and the arginine, something which will determine the pH under which the electrostatic interaction would be optimally formed. As the pH increases to alkaline conditions, the Asp194-His222 salt bridge weakens as His222 loses its extra proton. Under these conditions, the mutant H222R was stabilised by an additional salt bridge compared to wild type enzyme. Despite that the H222R substitution increased *T*_*m*_ under alkaline conditions (pH 10), it led to a decrease in *T*_*m*_ at pH 5 by -1.7°C.

## Discussion

In our group we have elected to focus on closely related proteins for which sufficient information (evidence of expression, thermal stability and other enzymatic properties) is available. Because of this relatedness, we believe that sequence-properties relationships inside the boundaries of a given protein family can be used successfully for predicting or controlling properties on the basis of DNA sequences analysis. Lipolytic enzymes from the recently identified N’ subfamily shares a very high degree of residue identity, yet display differences in stability and other properties. Consequently, they appear as ideal subjects for investigating the role of salt bridge residues in determining stability in this particular enzyme family.

### A 15^th^ family of bacterial lipolytic enzymes

We proposed the 15^th^ family (family XV) which correspond to abH11.03 on the basis of a distinct phylogenetic cluster (< 32% with family XIII), (Figure S8), the architecture of the cap domain (α/β cap) compared with the three α-helices cap of Est30-like proteins and suggest its separation and identification of a subfamily named N’ on the basis of a distinctive salt bridges pattern and on the polarity of a conserved interloop salt bridge in the active site. Similarly a distinctive pattern is conserved among the Est30-like group forming a subfamily within family XIII (N subfamily).

The proposed N’ subfamily contain lipolytic enzymes, which are typically ~ 250 residues carboxylesterases or monoacylglycerol lipases [7]. Structure comparison showed that the residues of the binding pocket are well conserved among EstGtA2 and MGL H-257, suggesting that other residues have an impact on specificity or other enzymatic properties. N’ enzymes have an additional domain over the active site (similar to the lid of lipases); they process long chain monoacylglycerol, but do not hydrolyze or synthesize long-chain triglycerides. Hydrolysis of long-chain triglycerides is a particular property of true lipases (EC 3.1.1.3) for which more elaborated lid structures allow for opening at the lipid-water interface, a phenomenon called interfacial activation. The architecture of the cap domain for N’ enzymes involves residues 125 to 163 (Figures 2-4). The recently identified LipS enzyme which shares many features with N’ enzymes, also has a similar cap (named here α/β cap). Other α/β hydrolases from distantly related organisms exhibit similar architecture in the cap domain. Interestingly, a cap architecture similar to EstGtA2, MGL H-257 or LipS was found in the crystal structure of distant α/β hydrolases including Est1E from *Butyrivibrio proteoclasticus* (2WTM), *Vibrio harveyi* thioesterase (1THT), esterase Rv0045c from *Mycobacterium tuberculosis* (2P2M), *Actinida eriantha* carboxylesterase AeCXE1 (2O7R), and *Streptococcus pneumoniae* EstA (2UZ0).

The corresponding cap is not found in closely related Est30 from *G. starothermophilus* (PDB no. 1TQH). In this case the cap is formed by three α-helices (D1’ D2’ B1’), involving residues 123 to 157 (Figures 3 and 4). The cap (this cap version will be referred to as α cap) architecture of the *G. stearothermophilus* Est30 (1TQH), which involves three helices, is also found in several distantly related carboxylesterases including: *Staphylococcus aureus* MenH (2XMZ), *Escherichia coli* ybfF (3BF7) and BioH (1M33), *Streptomyces aureofaciens* bromoperoxidase (1BRO), *Amycolatopsis mediterranei* thioesterase (3FLA) and *Agrobacterium radiobacter* epoxyde hydrolase (1EHY). Cap structure comparison supports the divisions proposed in phylogenetic analysis (Figure 5).

We found here that on the basis of the conserved salt bridges content, the cap structure, and on the polarity of the interloop salt bridge, N’enzymes clearly distinguishes from the other members of abH11.03 (or proposed family XV). LipS properties are shared by enzymes from both N’ and N subfamilies: it has the α/β cap typical for N’, but it has the interloop polarity of N (family XIII) and lacks the five bridges exclusive to N’. On the basis of our analysis of available sequences it appears that the N cluster includes Est30-like enzymes from family abH11.01 [9]. Similarly to N’, the N cluster forms a subgroup based on a distinctive salt bridges pattern.

Based on an earlier classification of α/β hydrolase enzymes (Lipase Engineering Database) as suggested by Fischer and Pleiss [9], carboxylesterases are grouped in the abH11 family. In this classification, the carboxylesterase Est30 (1TQH) belongs to abH11.01 while EstGtA2 belongs to the abH11.03 subfamily (Figure 5). In addition, the recently suggested LipS family [8], appears to belong to abH11.03. Therefore, the abH11.01 family (Fischer) and XIII [4] appear to be equivalent. The N’ cluster might be considered as a subfamily of abH11.03 (family XV). We propose to extend the Arpigny and Jaeger’s classification by introducing the new family XV (corresponding to abH11.03). The assignment of various clusters using the various classification schemes mentioned here is shown in Figure 6. The particular and exclusive salt bridges content and specific polarity of the interloop salt bridge are characteristics of the N’ subfamily.

### Role of key salt bridges in thermostability of EstGtA2

The demonstration that thermal denaturation (and its reversibility) was pH-dependent for EstGtA2 strongly supports a role for ionisable side chains in its stabilisation [19,20]. Thus, we chose to explore the role of arginine and lysine side-chains involved in the formation of the five conserved salt bridges in the N’ subfamily. Alanine scanning was chosen among other methods because we wanted to use a combinatorial approach, which cannot be reconciled with sophisticated approaches such as double-mutant cycle analysis (DMC) [45,46]. The substitution of alanine for arginine or lysine can be compared to “shaving” the side-chains. In this respect, one has to keep in mind that “alanine-shaving” of a particular side-chain removes electrostatic interactions as well as hydrophobic packing [47,48]. Every alanine substitution at Arg of Lys residues involved in salt bridges was found to decrease the stability of EstGtA2 at high temperature. The decrease in *T*_*m*_ was generally more pronounced for multiple mutations than for single ones. In all cases except one, folding and activity were observed at room temperature.

We show that when a particular residue is mutated (R37A), low temperature activity is increased, which might be due to change in enzyme flexibility, and concomitant favouring of higher activity at lower temperature [13]. This mutation also leads to shift the optimal temperature from 50 to 55°C compared to wild type. Moreover, this mutation was found to confer activity on long-chain triglycerides. Hydrolysis of long-chain triglycerides was detected for every mutant containing the R37A mutation. The mechanism by which this mutation changes specificity is under investigation.

Removal of bulky Arg led to limited changes in EstGtA2 tertiary structure packing, as indicated by near UV CD. Results from near and far-UV CD and fluorescence measurements indicate that EstGtA2 accommodated Ala substitutions without major structure modification in all but the M4c mutant. Thus, if we exclude the mutant M4c from the analysis, the five bridges or residues mutated do not appear to play a key role in specifying folding. It appears that their accumulation, and not their specific location or interactions, lead to loss of EstGtA2 stability and prevent correct folding of the enzyme in the case of M4c. The estimated conformational stability (Δ*G*_25°C_) for the wild type EstGtA2 (while partly reversible) at pH 8 is 11.8 kcal/mol, while for M4a is 4.9 kcal/mol. Despite a considerable reduced stability (ΔΔG_25°C_ = -6.9 kcal/mol), M4a remains active at low temperature. In the case of M4c, it appears that the ability of EstGtA2 to accept mutations of large and positively charged residues has been exceeded. What exact mutation(s) led to loss of compactness and activity at room temperature for this mutant is not clear. A similar conclusion was reached for indole-glycerol-phosphate synthase [12].

The extent to which the P22 Arc repressor can tolerate alanine substitutions has been reported. Interestingly, twenty five neutral positions were found and among these, the combination of 15 multiple-alanine substitutions can be tolerated without changing its conformation and/or general properties. However, the majority of the positions that do not tolerate alanine substitution involve residues forming the hydrophobic core of the protein or forming buried hydrogen bonds and salt bridges [49-52]. It was also reported that two buried salt bridges have a considerable impact in the folding pathway and the stability of Barnase [53,54]. This suggests that a limited number of the residues of a protein may determine its folding and its stability. Although that multiple salt bridges are directly related to thermostability of proteins from thermophiles, it is not clear how and to which extent they can play a determinant role in the folding process [55,56]. Distinct patterns of salt bridges were observed in closely-related proteins of the same fold. The role that may play these different salt bridge patterns in influencing protein folding and/or stabilizing the native conformation need to be explored.

The second part of this study focused on the interloop salt bridge D194-H222 (in EstGtA2). This particular bridge is found in N,N’families and in some lipolytic enzymes that have not been assigned to any of the families of the Arpigny and Jaeger classification (I to XIV). In particular we focused on His222 and the possible importance of p*K*_a_ value in determining pH dependence of enzymatic properties. As suggested by multiple alignments and phylogeny presented here, this bridge is very tolerant to mutation and accepted all five “usual” pH-responsive salt bridge-forming residues (Arg, Lys, His, Glu and Asp). Furthermore, the bridge can have any polarity in distantly related enzymes: in the N’ subfamily it is in opposite orientation compared to the corresponding bridge for enzymes from the N subfamily and close homolog LipS. The residue identity is switched in some cases. Considering this apparent flexibility and possible importance for enzymatic properties, we replaced the His residue found at position 222 in EstGtA2 for Arg. We found that His provided more stability at pH 5 than Arg at the same position. The fact that Arg did not confer the same stabilisation that His at the same position did below pH 5 might be explained by the different contributions of side-chain non-ionic interactions with the protein, as discussed previously [17]. Nevertheless, at pH 10, Arg provided a three-fold improvement in thermostability at 60°C. The high p*K*_a_ of Arg residue made that it should remain positive at higher pH, and thus maintain the coulombic interaction with D194. Is the proximity of the active site the explanation for this impact remains to be demonstrated, but clearly, the interloop salt bridge, common to families N (XIII or abH11.01) and N’ (XV or a sub-classification of abH11.03) is a determinant of thermostability.

## Conclusion

We identified five salt bridges that are a hallmark of lipolytic enzymes from the N’ subfamily (family XV), suggested by Charbonneau et al. 2010. We showed that the five positively charged residues involved in these bridges provide important stability and are essential for activity at high temperature. These key salt bridge-forming residues can be used for fine-tuning activity-temperature relationship. Despite their strict conservation in enzymes from the N’ subfamily, up to four salt bridge disruptions can be tolerated before EstGtA2 becomes unable to adopt its native fold. We also identified co-evolved residues within family XV and XIII that form a particular interloop salt bridge near the active site. Mutation at this interloop position in EstGtA2 (D194/H222), changing the His residue at position 222 for Arg, improved enzyme thermostability several fold under alkaline pH. Our study suggests primary targets for the optimization of EstGtA2 and enzymes from the N’ subfamily for specific biotechnological applications. These conserved salt bridge-forming residues may be useful sequence indicators for the assignation of newly discovered N’-related enzymes in the future.

## Supporting Information

Figure S1 **Structural model of EstGtA2.** **Structural alignment of EstGtA2 model and X-ray crystal structure of MGL H-257 (left) and with a 90° rotation (right).** Conserved salt bridges studied are shown. Conserved residues of the binding site are shown in orange.
(TIF)

Figure S2 **Amino acids substitution between EstGtA2 and MGL H-257.** The 27 out of 249 residues that are different in EstGtA2 compared to MGL H-257 (89% identity) are shown in red (EstGtA2) and orange (MGL H-257).
(TIF)

Figure S3 **Refinement statistics for the EstGtA2 model.** Ramachandran plot for the EstGtA2 model based on the X-ray crystal structure of MGL H-257 (PDB no. 3RM3).
(TIF)

Figure S4 **The E3-R54 salt bridge.** The first 86 residues from the N-terminal end are shown. The E3-R54 salt bridge links the N-terminal end of EstGtA2 to the core. A hydrogen bond between the R54 guanidinium and the oxygen of H58 and A11 backbone is predicted. In addition the R54 side-chain would form a hydrophobic cluster with two prolines (P13 before strand β2 and P33 after helix α1).
(TIF)

Figure S5 **Salt bridges interactions.** The predicted interactions for the following salt bridges: E3-R54 and E12-R37 (A), the E66-R140 (B), D124-K178 (C), D205-R220 and D194-H222 (D).
(TIF)

Figure S6 **Unfolding free energy for EstGtA2 and mutants.** The unfolding free energy (*ΔG*) as function of temperature (K) is shown. The melting temperatures (*T_m_*) are determined at *ΔG* =0.
(TIF)

Figure S7 **Hydrolysis of long-chain triglycerides by R37A.** The wild type EstGtA2 and mutants were deposited (10 µg) onto emulsified olive oil-agar plate containing rhodamine 0.001%. The hydrolysis of TAG released free fatty acids and the activity was detected under UV-illumination at 302 nm.
(TIF)

Figure S8 **The new family XV.** Phylogenetic tree showing the relationship between identified bacterial lipolytic enzyme families (I-XV). The new 15^th^ family is shown in bold.
(TIF)

File S1 **Supplementary tables.** Table S1, Primers used for cloning and directed mutagenesis. Table S2, Salt bridges studied. Table S3, Distinctive salt bridges composition between the N’, LipS and N clusters.
(PDF)
