# Correction: Role of Key Salt Bridges in Thermostability of *G*. *thermodenitrificans* EstGtA2: Distinctive Patterns within the New Bacterial Lipolytic Enzyme Family XV

**DOI:** 10.1371/journal.pone.0136940

**Published:** 2015-08-25

**Authors:** David M. Charbonneau, Marc Beauregard

After communicating with the corresponding authors of “ESTHER, the database of the α/β-hydrolase fold superfamily of proteins: tools to explore diversity of functions” [2], published in *Nucleic Acids Research*, the authors request to change the assignment of a new “family XV” described in our paper to an assignment of a new “subfamily XIII.2”.

At the time of publication, “family XV” had already been proposed as an extension of the original classification of Arpigny and Jaeger for a different family of enzymes by Lenfant, et al, in 2012. We would not have used the name “family XV” if we had been aware of the other study before publishing our work.

Based on extensive phylogenetic analyses, and structural and functional criteria, it is more appropriate to subdivide the family XIII. Every instance of “family XV” in the paper should be replaced by “subfamily XIII.2”

The correct title should be: Role of Key Salt Bridges in Thermostability of G. thermodenitrificans EstGtA2: Distinctive Patterns within the New Bacterial Lipolytic Enzyme Subfamily XIII.2. The correct citation should be: Charbonneau DM, Beauregard M (2013) Role of Key Salt Bridges in Thermostability of G. thermodenitrificans EstGtA2: Distinctive Patterns within the New Bacterial Lipolytic Enzyme Subfamily XIII.2. PLoS ONE 8(10): e76675. doi:10.1371/journal.pone.0076675

We also provide a revised Fig 5 and S8 Fig incorporating the new nomenclature.

**Fig 5:**
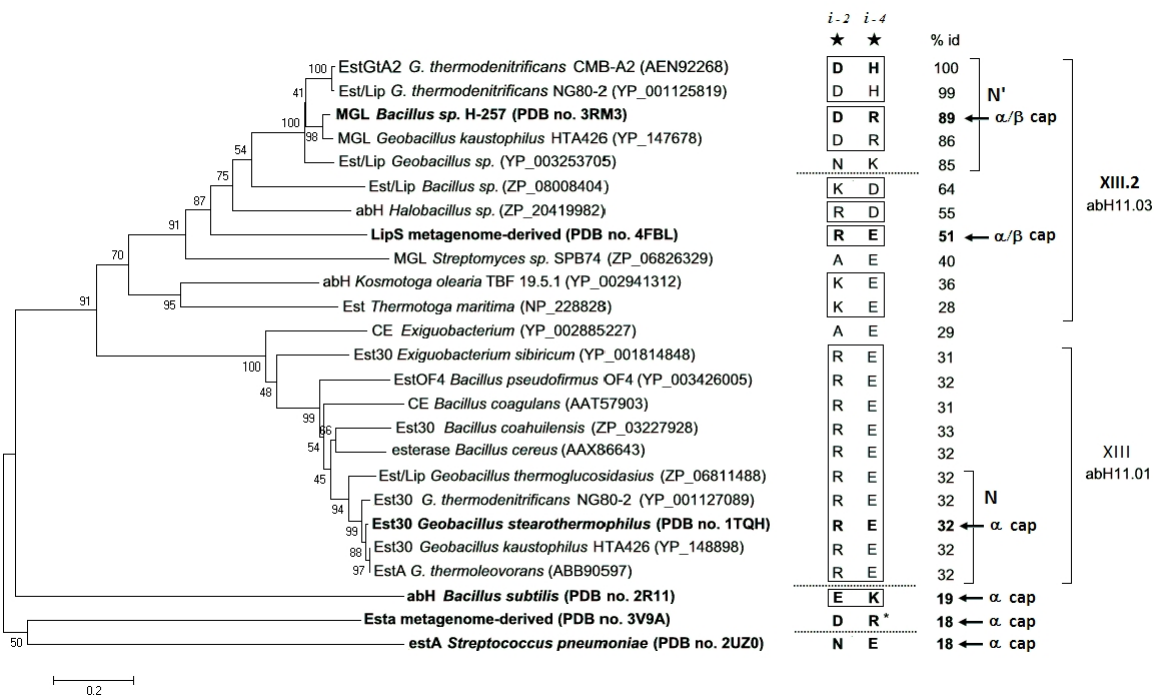
Evolution of the interloop salt bridge near the active site. A phylogenetic analysis of bacterial lipolytic enzymes related to subfamily XIII.2 and XIII on the separation of the N’ and N subfamilies. Sequences with solved crystal structures displaying the interloop salt bridge located in (i -2, i -4) from the catalytic Asp and His respectively are shown in bold. The corresponding ion pairs are shown right to the tree and annotated with a star and position relative to catalytic residues. Numbers show percentage identity compared with EstGtA2. Dashed lines indicate polarity reversals observed at the conserved interloop salt bridge position. The cap structure is indicated for X-ray resolved lipolytic enzymes. Sequences were assigned to N,N’ and abH11 families (taking into account enzymes classification in Lipase Engineering Database). The phylogenetic and molecular evolutionary analyses were conducted using MEGA version 5. The evolutionary history was inferred using the neighbor-joining method and the evolutionary distances were computed using the Poisson correction and are in the same units of the number of amino acid substitutions per site.

The criteria for the separation and assignation of protein sequences to the new subfamily XIII.2 are the same as the criteria described for the separation of the initially proposed family XV. The criteria proposed for the subdivision N’ are maintained identical to the initial proposition.

Criteria for the assignation of lipolytic enzymes to the family XIII.2:

- The enzymes belonging to subfamily XIII.2 show less than 40% sequence identity with enzymes belonging to the family XIII despite a high structural similarity of the α/β core domain.
- In contrast to the high structural similarity of the α/β core domain between enzymes belonging to the family XIII and subfamily XIII.2, lipolytic enzymes belonging to the subfamily XIII.2 exhibit a different architecture of the cap domain (type α/β) compared with enzymes from family XIII (type α).

Criteria for the assignation of lipolytic enzymes to the N’ cluster (a subdivision of subfamily XIII.2):

- The lipolytic enzymes belonging to the N’ cluster in the subfamily XIII.2 exhibit the described conserved interloop salt bridge located at position *i*-2 and *i*-4, from the catalytic Asp and His respectively, in a polarity of interaction (- / +), compared with a polarity of interaction (+ /-) for other enzymes included in the subfamily XIII.2 and for enzymes included in the family XIII.
- The lipolytic enzymes belonging to the N’ cluster in the subfamily XIII.2 show a conserved salt bridges pattern which comprises the following salt bridges: E3/R54, E12/R37, E66/R140, D124/K178 and D205/R220 (numbering based on the EstGtA2 sequence).

Functional criteria:

It is not clear how different the enzymes of the family XIII and XIII.2 are in terms of function or substrate specificity. However, the enzymes included in the proposed family XIII.2 display different types of hydrolytic activities. The three principal activities detected for enzymes of the XIII.2 are carboxylesterase (EC 3.1.1.1), esterase/lipase (EC 3.1.1.3) and monoacylglycerol lipase (EC 3.1.1.23). However, the complete set of hydrolytic activities for enzymes of the family XIII.2 and XIII is not known and does not allow for distinguishing them based on particular substrate specificity. Therefore, the principal criteria for the separation are based on the level of sequence identity and on the structural data, and not on functional data. The large structural difference in the cap insertion domain is likely to play a relevant role in function, but this aspect has not been investigated yet.

## Supporting Information

S8 Fig The new subfamily XIII.2. Phylogenetic tree showing the relationship between identified bacterial lipolytic enzyme families (I-XIII.2). The new subfamily XIII.2 is shown in bold.
(JPG)

## References

[1] Charbonneau DM, Beauregard M (2013) Role of Key Salt Bridges in Thermostability of G. thermodenitrificans EstGtA2: Distinctive Patterns within the New Bacterial Lipolytic Enzyme Family XV. PLoS ONE 8(10): e76675 doi:10.1371/journal.pone.0076675 24116134 10.1371/journal.pone.0076675 PMC3792869

[2] Lenfant N., Hotelier T., Velluet E., Bourne Y., Marchot P., & Chatonnet A. (2013). ESTHER, the database of the α/β-hydrolase fold superfamily of proteins: tools to explore diversity of functions. Nucleic Acids Research, 41(Database issue), D423–D429. doi:10.1093/nar/gks1154 23193256 10.1093/nar/gks1154 PMC3531081

