# Supplementary figures and images for: Role of Key Salt Bridges in Thermostability of G. thermodenitrificans EstGtA2: Distinctive Patterns within the New Bacterial Lipolytic Enzyme Family XV
Source: PLoS One. 2013 Oct 8;8(10):e76675. doi: 10.1371/journal.pone.0076675 (PMC3792869; doi:10.1371/journal.pone.0076675)

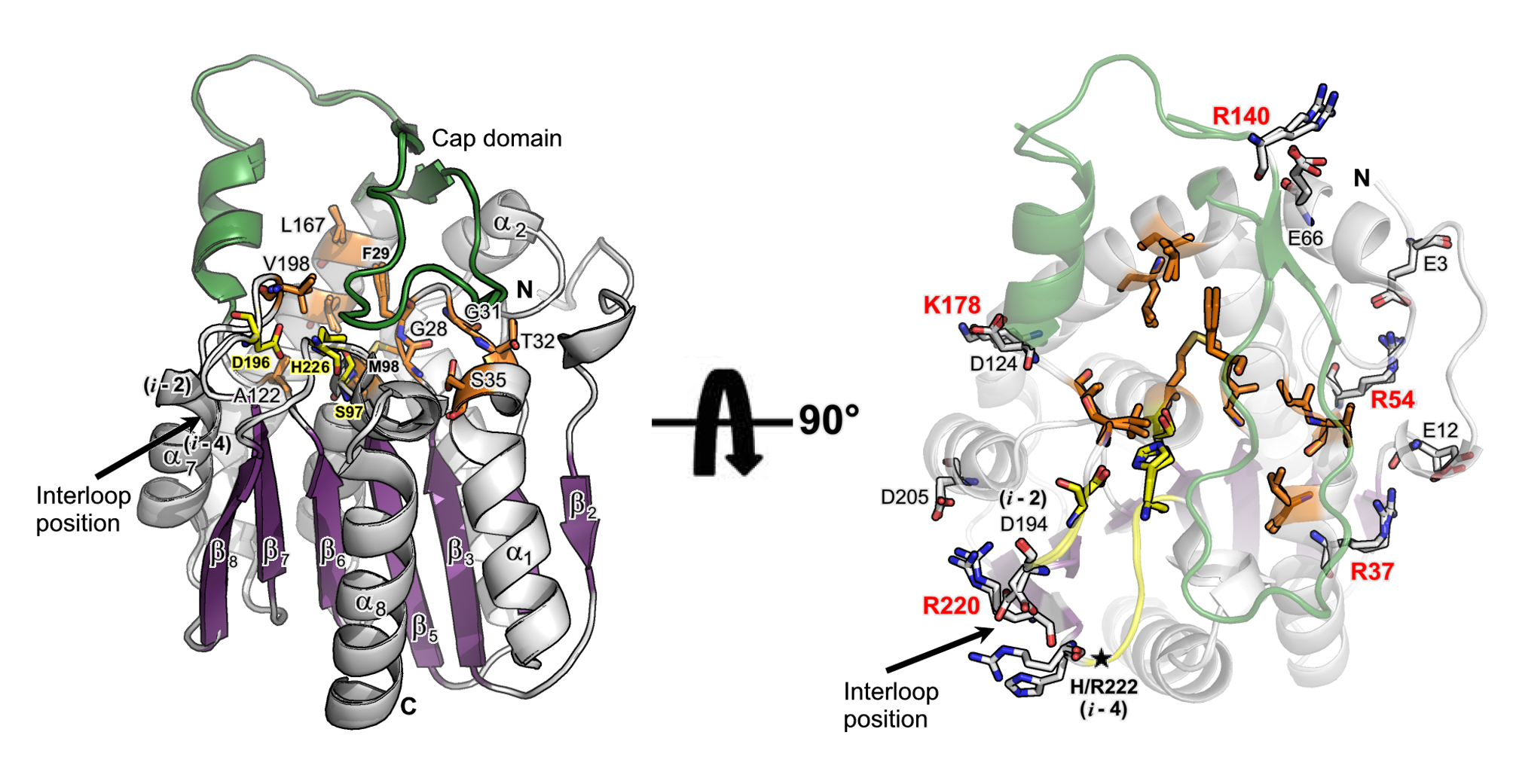

Supplement: Figure S1 — Structural model of EstGtA2. Structural alignment of EstGtA2 model and X-ray crystal structure of MGL H-257 (left) and with a 90° rotation (right). Conserved salt bridges studied are shown. Conserved residues of the binding site are shown in orange. (TIF) [file pone.0076675.s001.tif]

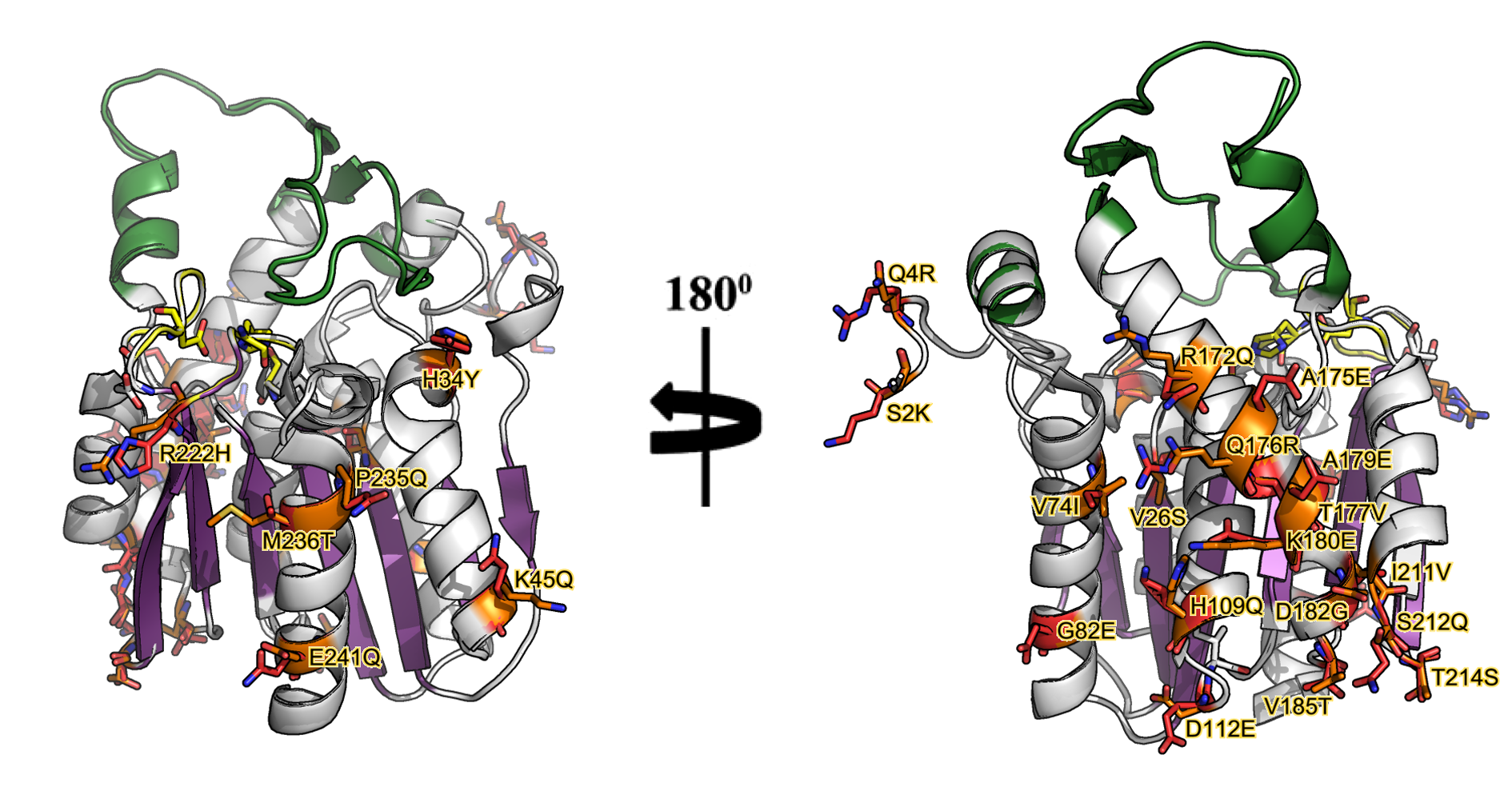

Supplement: Figure S2 — Amino acids substitution between EstGtA2 and MGL H-257. The 27 out of 249 residues that are different in EstGtA2 compared to MGL H-257 (89% identity) are shown in red (EstGtA2) and orange (MGL H-257). (TIF) [file pone.0076675.s002.tif]

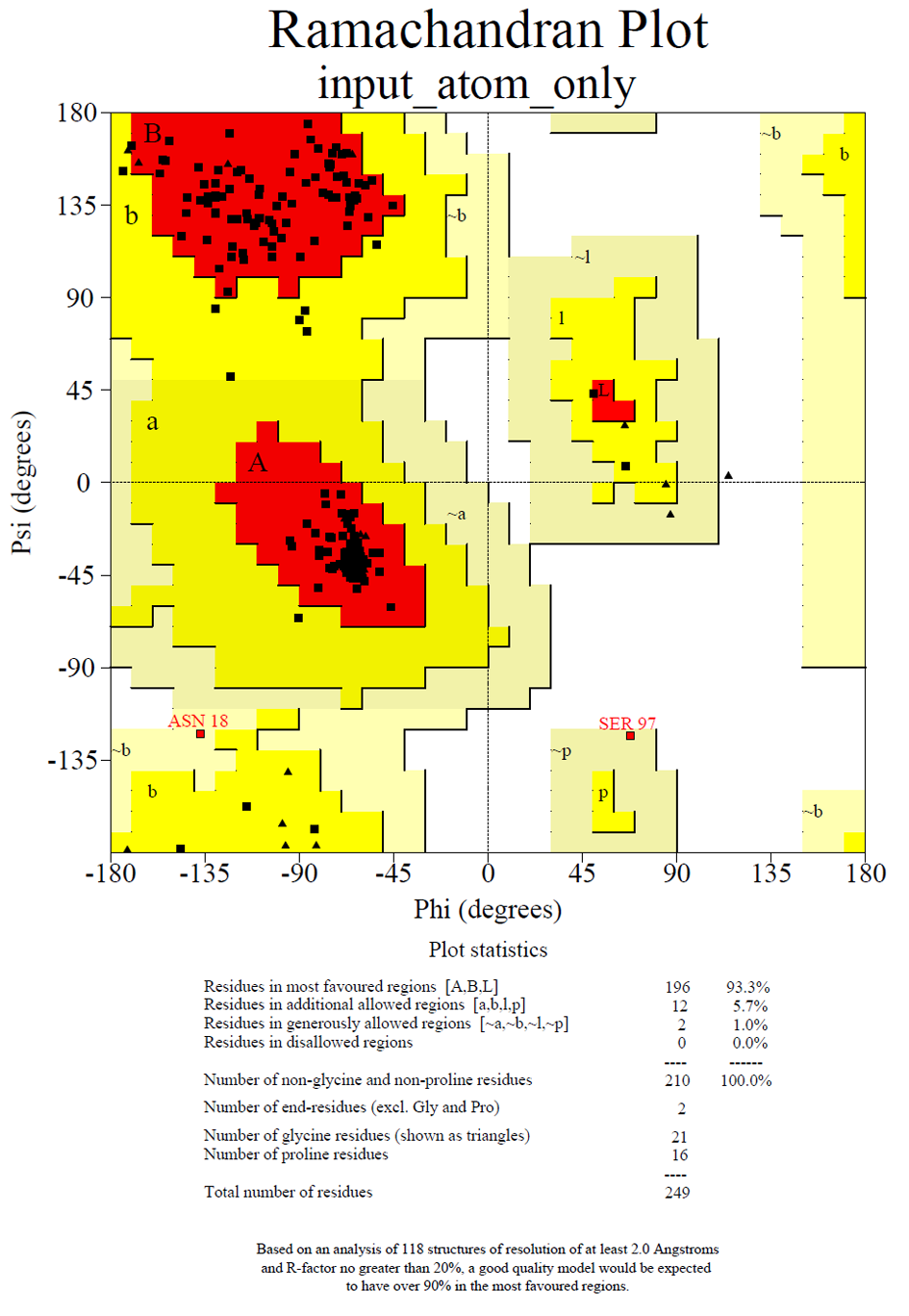

Supplement: Figure S3 — Refinement statistics for the EstGtA2 model. Ramachandran plot for the EstGtA2 model based on the X-ray crystal structure of MGL H-257 (PDB no. 3RM3). (TIF) [file pone.0076675.s003.tif]

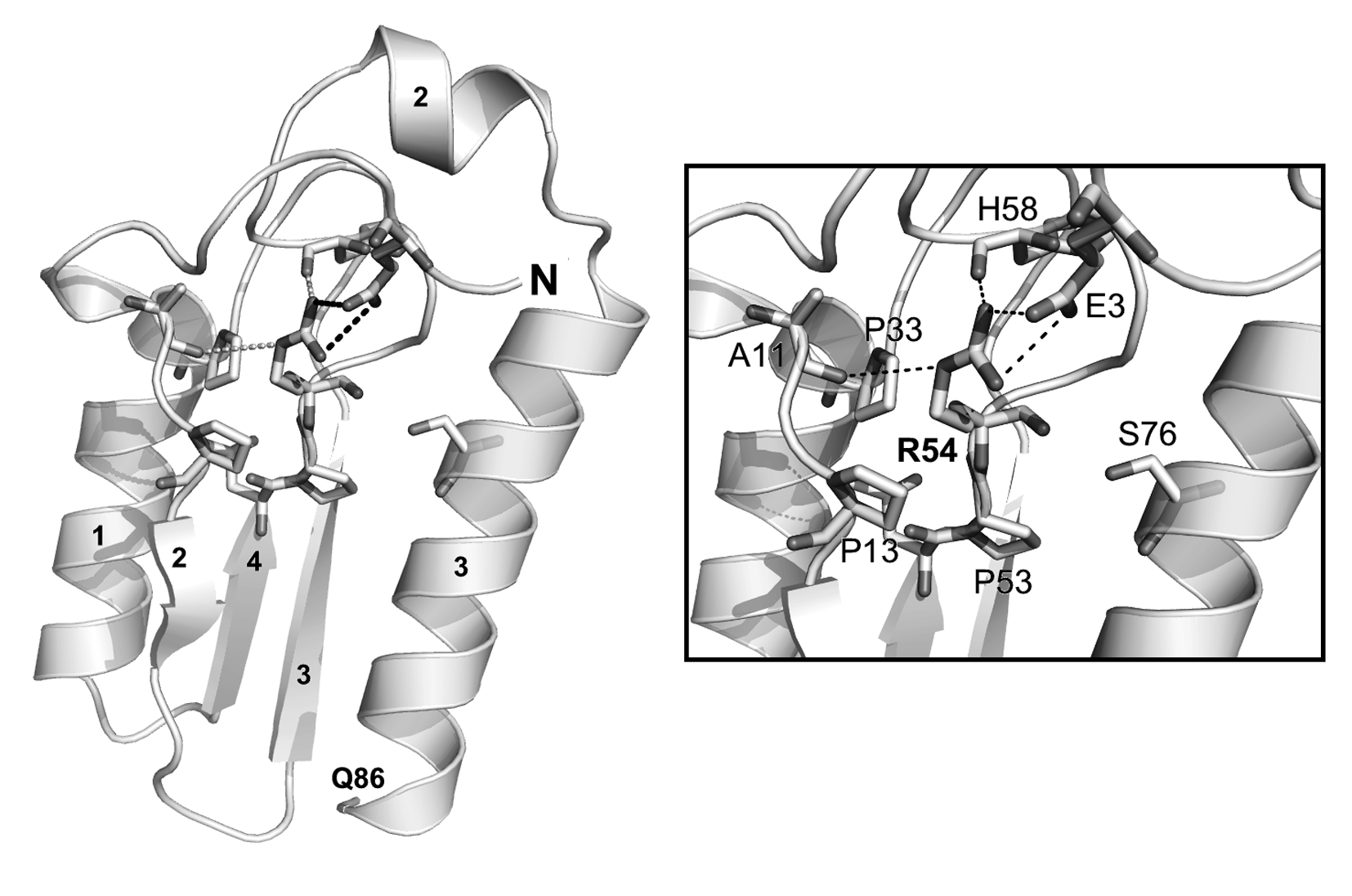

Supplement: Figure S4 — The E3-R54 salt bridge. The first 86 residues from the N-terminal end are shown. The E3-R54 salt bridge links the N-terminal end of EstGtA2 to the core. A hydrogen bond between the R54 guanidinium and the oxygen of H58 and A11 backbone is predicted. In addition the R54 side-chain would form a hydrophobic cluster with two prolines (P13 before strand β2 and P33 after helix α1). (TIF) [file pone.0076675.s004.tif]

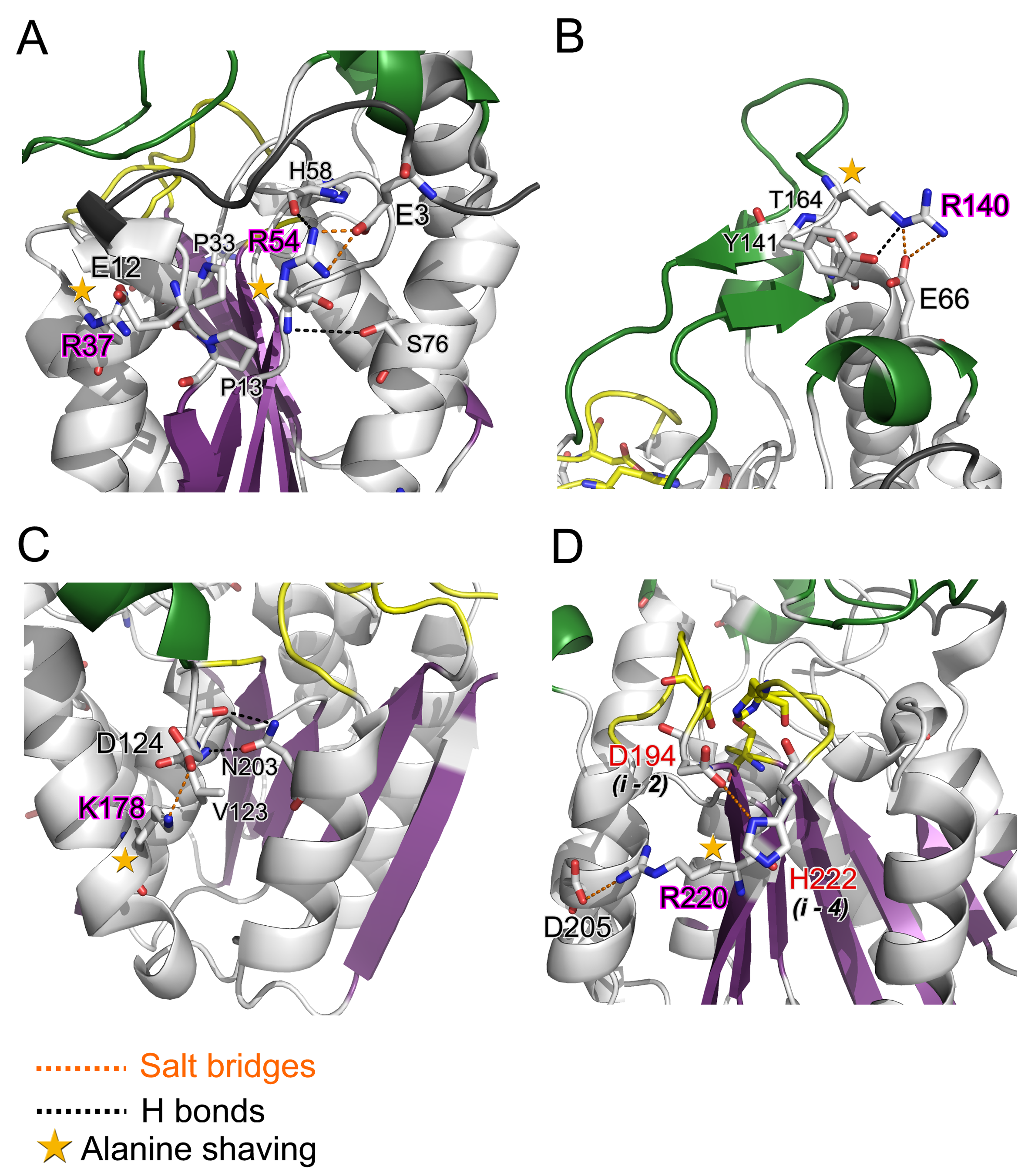

Supplement: Figure S5 — Salt bridges interactions. The predicted interactions for the following salt bridges: E3-R54 and E12-R37 (A), the E66-R140 (B), D124-K178 (C), D205-R220 and D194-H222 (D). (TIF) [file pone.0076675.s005.tif]

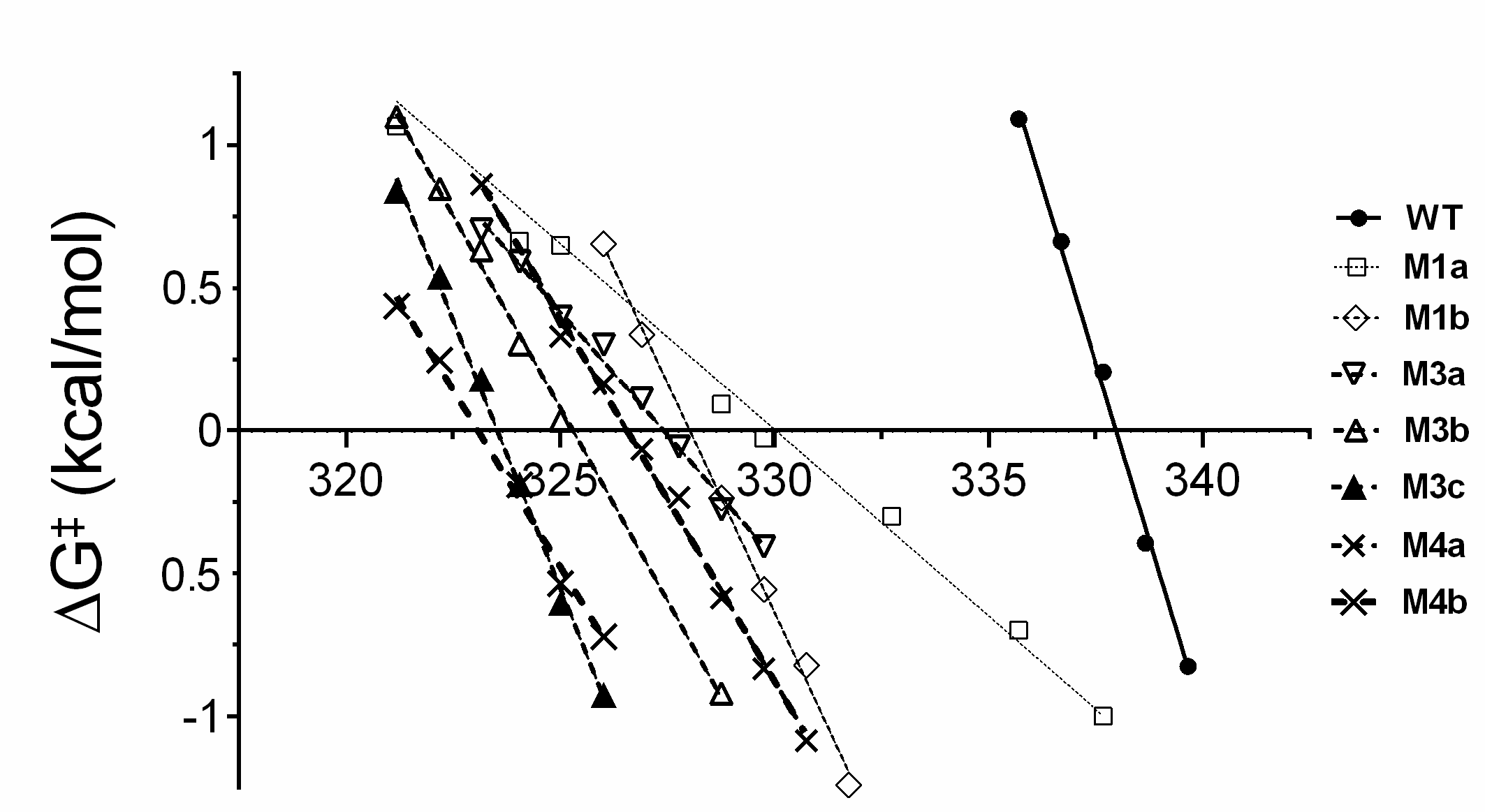

Supplement: Figure S6 — Unfolding free energy for EstGtA2 and mutants. The unfolding free energy (ΔG) as function of temperature (K) is shown. The melting temperatures (Tm) are determined at ΔG =0. (TIF) [file pone.0076675.s006.tif]

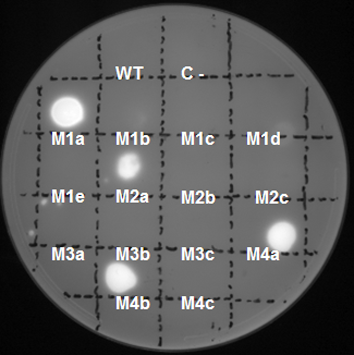

Supplement: Figure S7 — Hydrolysis of long-chain triglycerides by R37A. The wild type EstGtA2 and mutants were deposited (10 µg) onto emulsified olive oil-agar plate containing rhodamine 0.001%. The hydrolysis of TAG released free fatty acids and the activity was detected under UV-illumination at 302 nm. (TIF) [file pone.0076675.s007.tif]
