# Supplementary material for: Role of Key Salt Bridges in Thermostability of G. thermodenitrificans EstGtA2: Distinctive Patterns within the New Bacterial Lipolytic Enzyme Family XV
Source: PLoS One. 2013 Oct 8;8(10):e76675. doi: 10.1371/journal.pone.0076675 (PMC3792869; doi:10.1371/journal.pone.0076675)
Supplement: File S1 — Supplementary tables. Table S1, Primers used for cloning and directed mutagenesis. Table S2, Salt bridges studied. Table S3, Distinctive salt bridges composition between the N’, LipS and N clusters. (PDF) [file pone.0076675.s009.pdf]

File S1. SUPPLEMENTARY TABLES

Table S1. Primers used for cloning and directed mutagenesis

Primers used for cloning		Sites / Vector	Reference
5' TAATTAGGTACCTATGAAAGAACGATATCCTGTACTT 3'	Fwd	*Kpn*I / pQE31	Ref 7.
5' TATTAAAGCTTTCAAGCATGTTTGGCGAA 3'	Rev	*Hind*III / pQE31	
5’ CTCGGTACATATGAAAGAACGATATCCTGTACTTC 3’	Fwd	*Nde*I / pET28	This study
5’ GTGCGGCCGTCAAGCATGTTTGGCGAAAAACTG 3’	Rev	*Eag*I / pET28	
**Mutagenic primers**		**Mutation**	**Source**
5' CATACAGCATGGCGCCGCTTGCTGAAGCGTATG 3'	Fwd	R37A	This study
5' CATACGCTTCAGCAAGCGGCGCCATGCTGTATG 3'	Rev		
5' GTTATACCGTTTGCTTGCCGGCGTTAAAGGGGCATGGGACGC 3'	Fwd	R54A	This study
5' GCGTCCCATGCCCCTTTAACGCCGGCAAGCAAACGGTATAAC 3'	Rev		
5' TGGCGAGGTGCCGGCGTATCTTGACTCGAT 3'	Fwd	R140A	This study
5' ATCGAGTCAAGATACGCCGGCACCTCGCCA 3'	Rev		
5' CTCAATTGATGGAGCGTGTGGCGGAAGAGCTTGGACGCATTAC 3'	Fwd	K178A	This study
5' GTAATGCGTCCAAGCTCTTCCGCCACACGCTCCATCAATTGAG 3'	Rev		
5' CAGAGAAAGAGATCGTCGCGCTTCACAACAGCTACC 3'	Fwd	R220A	This study
5' GGTAGCTGTTGTGAAGCGCGACGATCTCTTTCTCTG 3'	Rev		
5' CTTTATTTGGCGGAACAGCGTCCGGAGATTTGCGGCATC 3'	Fwd	H110R	This study
5' GATGCCGCAAATCTCCGGACGCTGTTCCGCCAAATAAAG 3'	Rev		
5' GTTTCCGATGAAGACCGTGTTGTGCCGCCTGG 3'	Fwd	H197R	This study
5' CCAGGCGGCACAACACGGTCTTCATCGGAAAC 3'	Rev		
5’ GAGATCGTCCGTCTTCGTAACAGCTACCATGTAG 3’	Fwd	H222R	This study
5’ CTACATGGTAGCTGTTACGAAGACGGACGATCTC 3’	Rev		

Table S2. Salt bridges studied.

Acidic residues	Fraction accessible	Basic residues	Fraction accessible	Distance (Å)	Secondary structure
E3	0.18	R54	0.19	3.3	N-term/L5
E12	0.59	R37	0.20	3.9	L1/α1
E66	0.17	R140	0.74	3.6	αcap/Lcap
D124	0.40	K178	0.31	3.3	L10/α4
D205	0.59	R220	0.44	3.6	α5/β8
D194	0.52	H/R222	0.90/0.88	4.0/3.9	L14/L16

Table S3. Distinctive salt bridges composition between the N’, LipS and N cluster.

N'	LipS	N
E3-R54[a]	R66-E71	
E12-R37[a]	E114-R142	
E66-R140[a]	E115-R116	R37-E40
E78-H110	E141-K216	E124-K165
D124-K178[a]	R154-E209	K139-E152
D148-H197[b]	D179-H228[b]	E142-K144
**D194-H222**	**R225-E253**	**R191-E219**
D205-R220[a]	H233-E236	K216-D237
	E246-H279	
	E248-R271	

The conserved interloop salt bridge is shown in bold.

a. Salt bridges unique to N’ family enzymes.

b. Conserved salt bridge between EstGtA2 and LipS
