# Supplementary figures and images for: Correction: Role of Key Salt Bridges in Thermostability of G. thermodenitrificans EstGtA2: Distinctive Patterns within the New Bacterial Lipolytic Enzyme Family XV
Source: PLoS One. 2015 Aug 25;10(8):e0136940. doi: 10.1371/journal.pone.0136940 (PMC4549244; doi:10.1371/journal.pone.0136940)

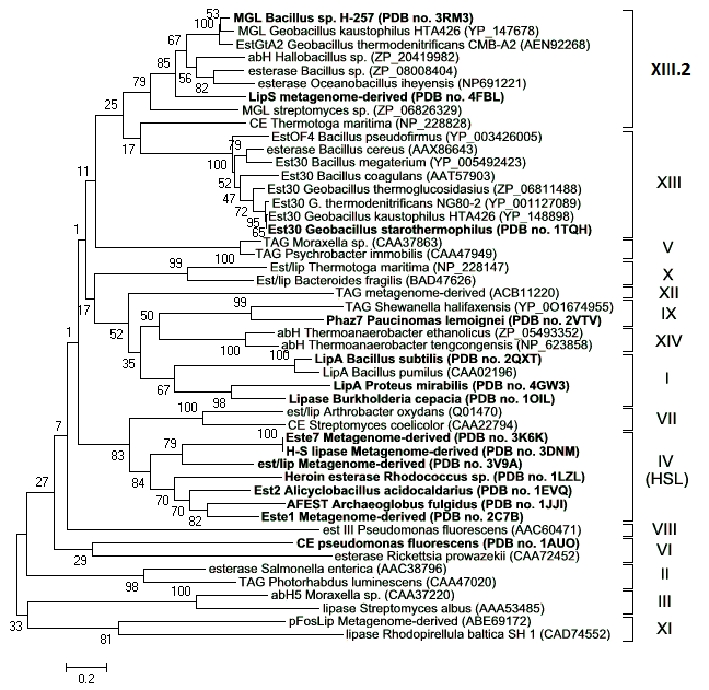

Supplement: S8 Fig — Phylogenetic tree showing the relationship between identified bacterial lipolytic enzyme families (I-XIII.2). The new subfamily XIII.2 is shown in bold. (JPG) [file pone.0136940.s001.jpg]
